# Cohesin Protects Genes against γH2AX Induced by DNA Double-Strand Breaks

**DOI:** 10.1371/journal.pgen.1002460

**Published:** 2012-01-19

**Authors:** Pierre Caron, Francois Aymard, Jason S. Iacovoni, Sébastien Briois, Yvan Canitrot, Beatrix Bugler, Laurent Massip, Ana Losada, Gaëlle Legube

**Affiliations:** 1 Université de Toulouse, UPS, LBCMCP, Toulouse, France; 2 CNRS, LBCMCP, Toulouse, France; 3 Bioinformatic Plateau I2MC, INSERM and University of Toulouse, Toulouse, France; 4 Chromosome Dynamics Group, Spanish National Cancer Research Centre (CNIO), Madrid, Spain; Institut Jean-Pierre Bourgin, INRA de Versailles, France

## Abstract

Chromatin undergoes major remodeling around DNA double-strand breaks (DSB) to promote repair and DNA damage response (DDR) activation. We recently reported a high-resolution map of γH2AX around multiple breaks on the human genome, using a new cell-based DSB inducible system. In an attempt to further characterize the chromatin landscape induced around DSBs, we now report the profile of SMC3, a subunit of the cohesin complex, previously characterized as required for repair by homologous recombination. We found that recruitment of cohesin is moderate and restricted to the immediate vicinity of DSBs in human cells. In addition, we show that cohesin controls γH2AX distribution within domains. Indeed, as we reported previously for transcription, cohesin binding antagonizes γH2AX spreading. Remarkably, depletion of cohesin leads to an increase of γH2AX at cohesin-bound genes, associated with a decrease in their expression level after DSB induction. We propose that, in agreement with their function in chromosome architecture, cohesin could also help to isolate active genes from some chromatin remodelling and modifications such as the ones that occur when a DSB is detected on the genome.

## Author Summary

Genomic stability requires that deleterious events such as DNA double-strand breaks (DSBs) are precisely repaired. The natural compaction of DNA into chromatin hinders DNA accessibility and break detection. Therefore, cells respond to DSBs by triggering multiple chromatin modifications that promote accessibility and facilitate repair. We have recently developed a novel system whereby a restriction enzyme can be induced to inflict multiple DSBs across the human genome. This system permits high-resolution characterization of changes in the chromatin landscape that are induced around DSBs. While we previously reported the profile of H2AX phosphorylation (a primary event in chromatin remodelling that takes place in response to DSBs), we now provide the high resolution mapping of cohesin, a complex implicated in the 3-D organisation of chromosomes within the nucleus. Unexpectedly, we have discovered that cohesins play a role in the maintenance of gene transcription in regions where chromatin has been remodelled during the DSB response.

## Introduction

DNA packaging into chromatin hinders detection and repair of DNA Double Strand Breaks (DSBs), and therefore DSB repair occurs simultaneously with multiple chromatin modifications, including histone acetylation, ubiquitylation and phosphorylation, as well as ATP dependant nucleosome remodelling and chromatin protein deposition or exclusion (for review [1],[2]). These chromatin changes not only generate a chromatin state permissive to DNA repair, but also contribute to DSB signalling and checkpoint activation. Phosphorylation of H2A in yeast or H2AX in mammals (referred to γH2AX) occurs rapidly, within a few minutes, and is considered to be one of the first DSB-induced chromatin modifications. While γH2AX is not required for the initial recruitment of repair proteins onto DSBs, it is necessary for the proper assembly of repair foci (also called IRIF, for IRradiation Induced Foci) and full activation of the DNA Damage Response (DDR) [3], [4]. H2AX deficient mice are radio-sensitive and subject to increased genomic instability [5], highlighting the critical function of γH2AX *in vivo*. Remarkably, γH2AX spreads across large chromatin domains surrounding DSBs, around 50 kb in yeast [6] and up to 2 Mb in vertebrate cells [7]–[11]. Until recently, the mechanism(s) underlying such wide spreading, as well as its consequences on chromatin activity and gene transcription were unclear. Indeed, several lines of evidence indicated that DSB generation triggers RNA Pol II and Pol I exclusion/pausing at break sites and inhibits transcription of proximal genes in an ATM dependent manner [12], [13]. However, whether and how transcription was affected further distally from the break in γH2AX domains remained elusive [6], [14]. Recently, we developed a stable human cell line, designed for controlled, sequence-specific DSB induction, based on the expression of an 8 bp restriction enzyme (*Asi*SI) fused to the oestrogen receptor ligand binding domain. Using this system, we monitored γH2AX distribution and changes in transcription, around more than 20 DSBs located on chromosomes 1 and 6 using ChIP-chip [10].We uncovered that γH2AX spreads unevenly over megabases of surrounding chromatin, avoiding transcribed genes. Within γH2AX domains, we found that gene transcription remained unchanged upon DSB induction [10]. We suggested that the γH2AX profile reflects the spatial organisation of chromatin and proposed a 3-dimensional model, which accounts for the accurate maintenance of gene transcription proximal to DSBs via their exclusion outside of γH2AX foci.

In addition to γH2AX, evidence suggests that cohesin plays a critical role in DSB repair (for review [15], [16]). Cohesin is a multi-subunit complex, thought to embrace DNA as a ring-shaped structure, that mediates sister chromatin cohesion and ensures accurate chromosome segregation. It consists of the proteins SCC1 (also termed Rad21/Mcd1p), SCC3 (SA1 and SA2 in human somatic cells) and the heterodimer SMC3/SMC1. In yeast, cohesin is recruited over a 50 kb chromatin domain surrounding an HO-induced break [17]–[19]. In vertebrate cells, cohesins are targeted to chromatin upon ionizing radiation [20] and to DSBs induced by X ray stripes and laser tracks during G2 [21]–[23], although this may only occurs at very high power settings [22]. However, ChIP studies clearly showed that SMC1 and SCC1 are recruited to an I-SceI-induced DSB [24], suggesting that loading of cohesin at DSBs also occurs in mammalian cells. Cohesin promotes equal homologous recombination between sister chromatids and prevents homologous recombination between repeats or homologous chromosomes [24]–[28]. In addition, its function in DSB repair depends upon cohesion establishment, a phenomena known as DIC (Damage Induced Cohesion) ([29]–[32] for review [33]). This led to the proposal that cohesin may participate in post-replicative DNA repair by ensuring proper cohesion between sister chromatids thus facilitating homologous recombination with the sister locus.

Importantly, beyond its role in DSB repair and sister chromatid cohesion, another function for cohesin has recently emerged. In vertebrates, the cohesin complex accumulates at specific loci, mainly enhancer/promoters and sites bound by the CTCF insulator protein [34]–[36]. There, it participates in the transcriptional control of neighbouring genes, most likely through its ability to mediate long-range interactions between chromatin fibers, thereby allowing enhancer/promoter interaction and/or insulation from the surrounding chromatin [34]–[37]. More generally, cohesins are now believed to play a critical role in genome organization, participating in loop formation and thus affecting various DNA-based processes such as transcription and replication [38].

Given the multiple roles of cohesin in DSB repair, higher-order chromatin structure and transcriptional control, we decided to characterize the cohesin profile around *Asi*SI-induced DSBs in order to both further refine its function in DSB repair and its potential impact on γH2AX spreading. Here we show that, in contrast to yeast, cohesin is only moderately recruited to *Asi*SI-induced DSBs in human cells and does not spread over more than 5 kb. Remarkably, cohesin binding antagonizes γH2AX accumulation within γH2AX domains. Depletion of the SCC1 cohesin subunit leads to both an increase in γH2AX and a DSB-dependent transcriptional downregulation of genes within γH2AX domains, suggesting that cohesins are, at least in part, responsible for the accurate transcriptional control observed in γH2AX domains. Finally, we also analyzed the consequences of cohesin depletion on the positions of γH2AX domain boundaries, and found that while most of these boundaries remained unaffected, at some genomic locations cohesin helped to confine γH2AX spreading.

## Results

### Moderate and confined recruitment of cohesin at *Asi*SI-induced DSBs

**Figure 1:**
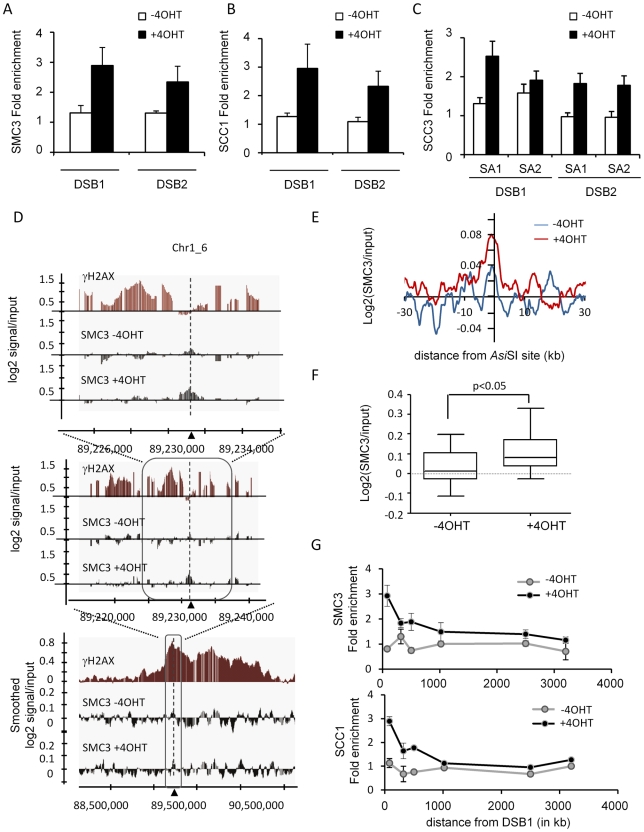
SMC3 is recruited locally around DSBs on the mammalian genome. A, SMC3 ChIPs were performed on *Asi*SI-ER-U20S cells, before and after 4 hours of 4OHT treatment and SMC3 enrichment was scored by Quantitative RealTime PCR (Q-PCR) in the vicinity (respectively 80 bp and 200 bp) of two *Asi*SI-induced DSBs as indicated. The fold enrichment relative to a genomic sequence devoid of *Asi*SI site (2 MB away from the closest site) is presented. Mean and Standard deviation of the mean (SDOM) of 4 independent experiments are shown. B, Same as in A, except that an SCC1 antibody (Abcam) was used. C, Same as in A, except that SA1 or SA2 antibodies were used. D, SMC3 ChIPs before and after 4 hours of 4OHT treatment were hybridized on Human Tiling Array 2.0R-A covering chromosomes 1 and 6. The log2 of the SMC3/input ratio is presented around one of the *Asi*SI sites (indicated by an arrow). The γH2AX profile previously characterized [10] is shown in red. Successive views are presented. For the widest view (bottom panel) the signals were smoothed using a sliding window size of 500 probes. Note that the SMC3 enrichment after 4OHT treatment is only detectable in the vicinity of the *Asi*SI site. E, Average SMC3 profile before (blue) and after (red) 4OHT treatment, plotted relative to *Asi*SI site positions. F, The average log2 (SMC3/input) over a 2000 bp window centered on DSB, for the 24 cleaved *Asi*SI sites of the chromosome 1 and 6 (see Table S1), was calculated before and after 4OHT treatment. The distribution is represented as a box plot. The *p* value (paired t-test) is indicated above. G, SMC3 and SCC1 ChIP were performed before and after 4OHT treatment and spreading was monitored by Q-PCR using primers pairs located respectively at 80 bp, 319 bp, 500 bp, 1019 bp, 2500 bp and 3200 bp away from the DSB1. The fold enrichments relative to a genomic sequence devoid of *Asi*SI site are plotted. A representative experiment is shown.

We recently developed a human cell line that stably expresses an *Asi*SI-ER fusion restriction enzyme (the *Asi*SI-ER-U20S cell line). Treatment with hydroxytamoxifen (4OHT) triggers nuclear localisation of the enzyme and induces DSBs at defined genomic loci, enabling ChIP analyses of protein recruitment at DSBs [10]. In order to better understand the function of cohesin in DSB repair, we thus performed ChIPs against various human cohesin subunits before and after break induction. The specificity of homemade antibodies was first confirmed using western blot, immunoprecipitation and ChIP assays on a known cohesin-binding site [35] (Figure S1 and Figure S2.). We found that 4OHT treatment induced the targeting of SMC3, SCC1 and SCC3 (SA1 and SA2) at *Asi*SI-induced DSBs (Figure 1A, 1B, 1C respectively) indicating that the full complex is likely to be recruited at DSBs.

Since it was previously reported that cohesins may target DSBs preferentially in the G2 phase of the cell cycle [21], we monitored SCC1 recruitment in G2 arrested *Asi*SI-ER-U20S cells following a RO-3306 treatment. We did not find a major difference in loading of SCC1 onto DSBs when compared with asynchronous cells (Figure S3A). In addition, we also used the *Asi*SI-ER-T98G cell line [10], [11] that can easily be synchronized by serum starvation, to monitor cohesin recruitment in G1 and G2 synchronized cells. Again, SCC1 DSB-targeting was similar in G1 and G2 (Figure S3B). A ChIP performed at 14 hour after 4OHT treatment ensured that SCC1 recruitment did not change drastically at a later time point (Figure S4).

We therefore decided to perform SMC3 ChIP-chip experiments in asynchronous cells, before and after 4 hours of 4OHT treatment, using human Affymetrix tiling arrays covering chromosomes 1 and 6, in order to simultaneously investigate the distribution of cohesins around multiple DSBs with high resolution. On these two chromosomes, the SMC3 distribution in untreated *Asi*SI-ER-U20S cells was similar to the distribution of SCC1 reported for HeLa cells [35] (see examples Figure S5A). 37.6% of SMC3 binding sites identified in *Asi*SI-ER-U20S were also identified using the SCC1 dataset from HeLa cells. Both SCC1 and SMC3 signals showed a clear enrichment at transcription start sites (TSS) (Figure S5B), consistent with the fact that a significant proportion of cohesin binding sites are located in close proximity to promoters [34]–[36], results which confirm the validity of our ChIP-chip data.

Strikingly, we found that recruitment of SMC3 at DSBs induced by 4OHT treatment was moderate and did not spread widely around the DSB to form a γH2AX-like domain, but rather localized within close proximity to the break (Figure 1D). When averaged around the 24 *Asi*SI-induced DSBs on chromosomes 1 and 6 ([10]; see Table S1 for a list of *Asi*SI sites), the SMC3 profile showed a weak increase upon 4OHT addition over a ∼5 kb region surrounding the DSB (Figure 1E). Although weak, we found that this increase of SMC3 after 4OHT treatment at the vicinity of *Asi*SI sites (on a 2 kb window) was significant (*p*<0.05) (Figure 1F and Figure S6).

In order to confirm that cohesin did not spread around DSBs in our cell line, we performed ChIP followed by Q-PCR analyses using primer pairs located at various positions from a DSB. Both SMC3 and SCC1 showed a clear increase upon 4OHT treatment at the immediate vicinity of the break, recruitment that was undetectable further away from the DSB (Figure 1G). Importantly, several labs previously reported an extended recruitment of cohesin over 50 kb domains around a single HO-induced DSB in yeast [17]–[19]. Since in our cell line, 4OHT treatment induced over a hundred DSBs [11], we wondered whether the lack of cohesin spreading observed here could be due to a limiting amount of free cohesin or/and available cohesin loaders, for targeting at DSBs. In order to address this point, we first controlled the amount of soluble cohesin (unbound to chromatin) in the nucleus before and after 4OHT treatment. Both SCC1 and SMC3 were still present in the soluble fraction after DSB induction (Figure S7A), indicating that free cohesins are not a limiting factor in these conditions. In addition, we also performed a SCC1 ChIP in an I-*Sce*I-ER U20S cell line, in which one single DSB is induced upon 4OHT treatment. As observed on *Asi*SI-induced DSBs, we could detect a 4OHT-dependant increase of SCC1 at the I-*Sce*I-induced DSB (300 bp), but not at 2.4 kb from the DSB (Figure S7B). Thus the high amount of DSBs induced by *Asi*SI over the human genome is not responsible for the lack of spreading observed in human cells.

Altogether, our data indicate that in human cells, cohesin is moderately targeted to DSBs and that it does not spread over wide chromosomal domains in contrast to yeast.

### Cohesin counteracts γH2AX spreading

**Figure 2:**
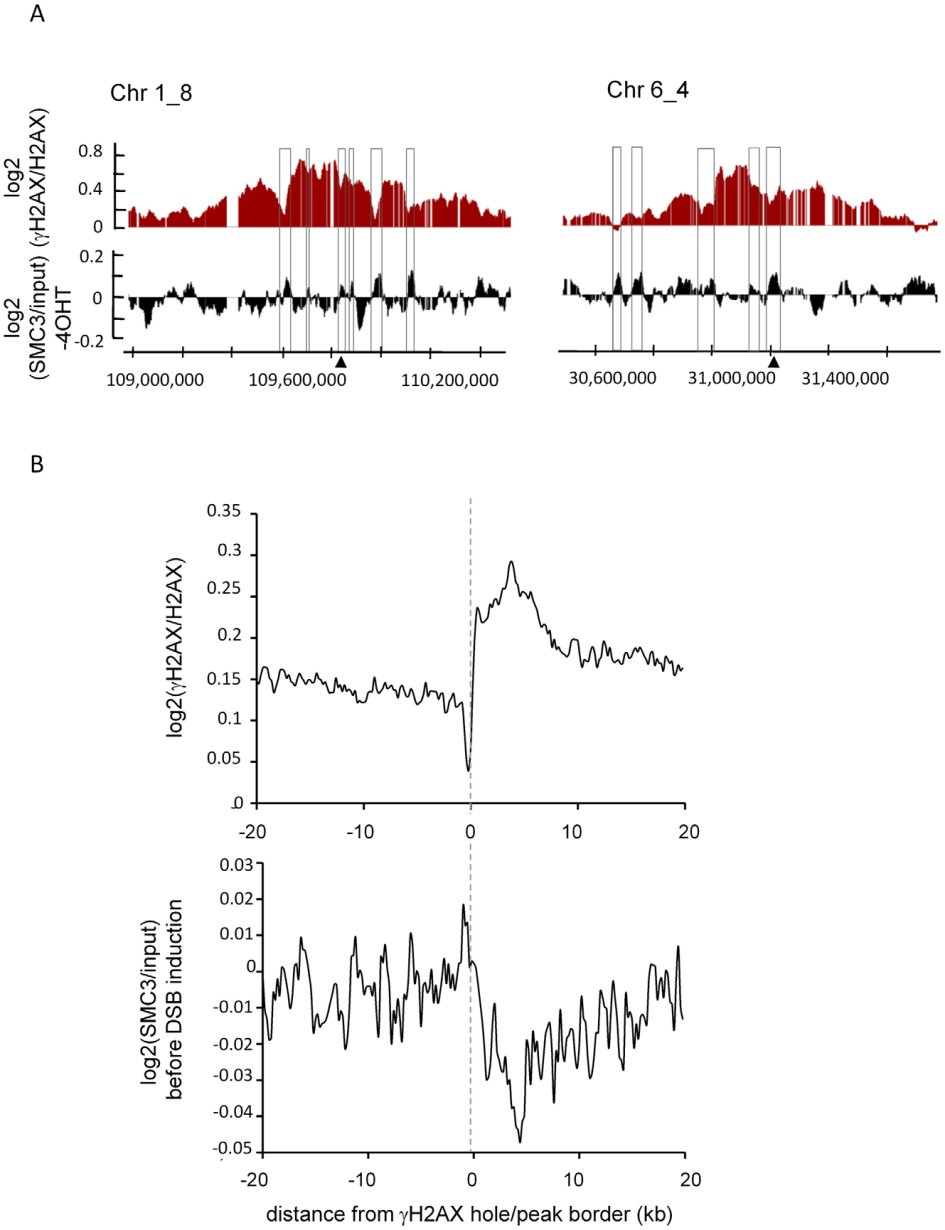
SMC3 counteracts γH2AX. A, Detailed views of the γH2AX/H2AX signal after 4OHT treatment (red) and the SMC3/input signal before 4OHT treatment (black) around two *Asi*SI sites (indicated by arrows), expressed as log2 and smoothed using a 500 probes sliding window. B, Regions depleted in γH2AX inside the γH2AX domains were identified using the algorithm detailed in [10] (applied on the average of two 4OHT-induced γH2AX/H2AX ChIP-chip studies). 534 “hole” borders were aligned and overlaid, right and mirror left borders are combined. Profiles are shown for γH2AX (Top) and SMC3 (Bottom) over a 40 kb window centered on the hole borders and averaged using a 200 bp window size. Note the enrichment of SMC3 in the γH2AX holes.

During the course of previous studies, we noticed that γH2AX within domains tended to decrease on cohesin peaks identified in HeLa cells [35] (Figure S8). Thus we next compared the cohesin distribution obtained in our *Asi*SI-ER U20S cells in absence of DSB induction, with our previously reported γH2AX profile. Within γH2AX domains, areas showing low levels of γH2AX (“holes”) often coincided with peaks of SMC3 monitored before 4OHT treatment (Figure 2A). We retrieved the γH2AX peak/hole positions within domains (see Material and Methods) and averaged the profile of SMC3 across their borders. γH2AX peak/hole transition coincided with a change in the SMC3 profile (Figure 2B). In addition, we also found that the genes showing high levels of SMC3 rather harbour low level of γH2AX (Figure S9). In order to confirm these data we also profiled SCC1 in our cell line under normal conditions. Again, the SCC1 distribution in *Asi*SI-ER U20S cells was similar to the profile characterized in HeLa cells (Figure S10A–S10B), and 43% of the binding sites in *Asi*SI-ER U20S cells, overlapped with binding sites in HeLa cells. We found that, as observed with SMC3, SCC1 peaks coincided with γH2AX holes, and that SCC1 rich genes showed low levels of γH2AX (Figure S11).

Altogether these results suggest that the cohesin present onto chromatin before any DSB induction antagonizes γH2AX establishment/maintenance.

**Figure 3:**
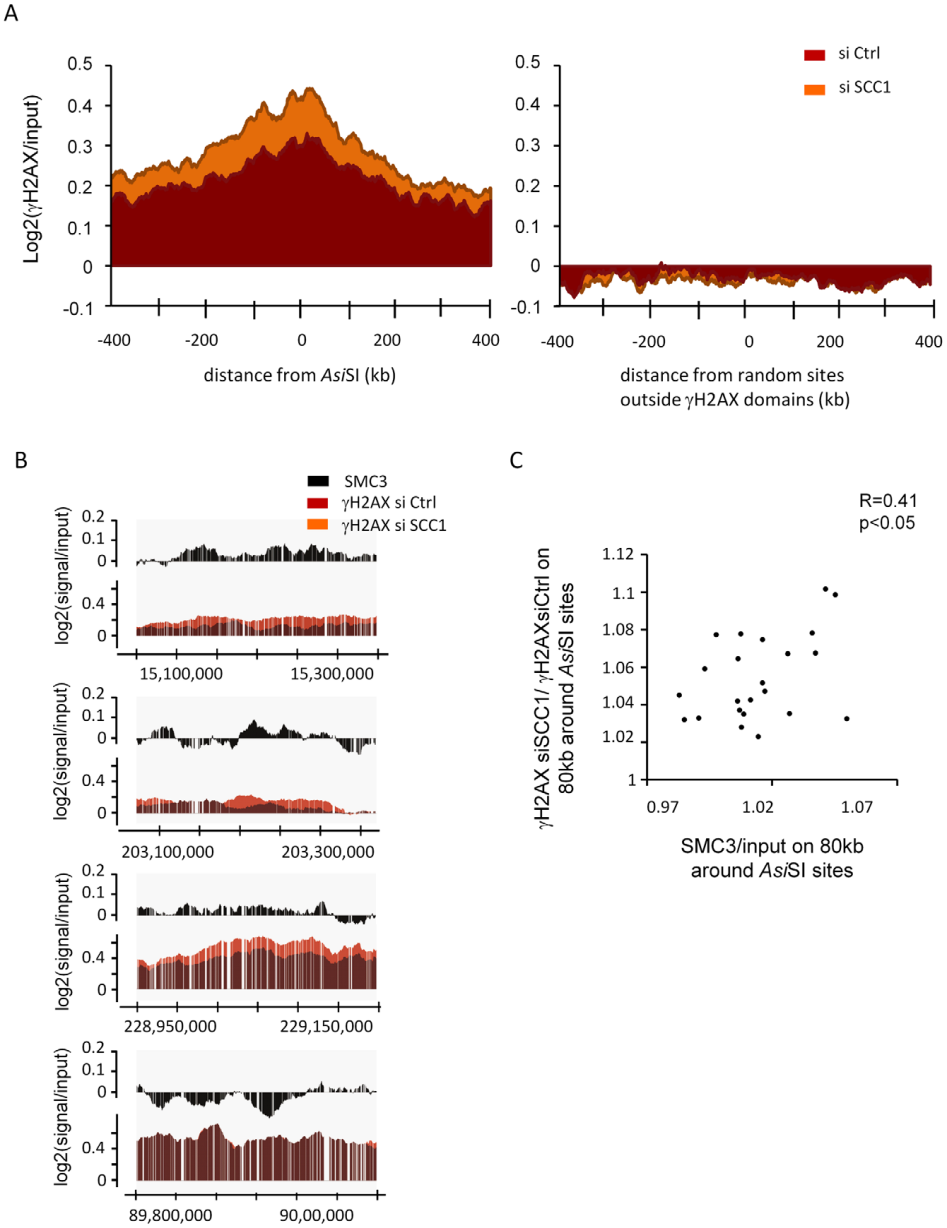
Cohesin depletion leads to an increase of γH2AX. A, γH2AX ChIP-chip were performed after 4OHT treatment in control (black) or SCC1 depleted (red) cells. The log2 γH2AX/input signal from two independent experiments was averaged around the 24 digested *Asi*SI sites from chromosomes 1 and 6 (see Table S1) (left panel), or on averaged around 24 random sites located outside γH2AX domains (right panel). B, Detailed views of the ChIP-chip signals (from two experiments) obtained for SMC3 (black), γH2AX in control cells (red) and γH2AX in cells treated with SCC1 siRNA (orange), on three cohesin-bound regions (upper and middle panels) and one cohesin-unbound region (lower panel). C, The averaged SMC3 signal over an 80 kb window around each *Asi*SI site was calculated (x-axis) and plotted against the γH2AX ratio in cells transfected with siRNA_SCC1 versus siRNA_CTRL (y axis). The correlation coefficient and the *p* value are indicated.

To test this hypothesis we analysed by ChIP-chip the γH2AX profile upon SCC1 depletion by siRNA. Depletion of this subunit has been shown to also trigger an almost complete disappearance of SMC3 from chromatin [35]. SCC1 siRNA [35] was highly efficient since both RNA and protein levels were strongly reduced (Figure S12A–S12B). In addition, chromatin-bound SCC1 was also efficiently depleted by siRNA as shown by ChIP (Figure S12C). We observed that within domains, γH2AX signals increased in SCC1 depleted cells when compared to cells transfected with control siRNA (Figure 3A left panel, Figure S13 upper and middle panels, and Figure S14A). This was also confirmed by Q-PCR analyses of γH2AX ChIP in control and SCC1 depleted cells (Figure S15), using primers pairs located at various positions from the DSB in five different γH2AX domains.

This increase was not detected elsewhere on the genome indicating that it was not due to an effect of SCC1 depletion on basal levels of γH2AX (Figure 3A right panel, Figure S13 lower panels and S14B). Our cleavage assay indicated that SCC1 depletion did not change the efficiency of *Asi*SI site cutting (Figure S16). Therefore, the enhanced phosphorylation of H2AX observed in SCC1 depleted cells was not due to an increase in *Asi*SI-ER activity, but rather to some modification(s) of the establishment or maintenance of γH2AX on chromatin. Importantly, we could also detect this increase by immunofluorescence (Figure S17), and changes in γH2AX levels upon SCC1 depletion have also been observed by western blot using irradiated cells [39], which further support our findings.

Furthermore, we found that the γH2AX increase observed upon SCC1 siRNA transfection occurred preferentially on cohesin-bound chromatin (Figure 3B). The ratio of γH2AX in SCC1-depleted versus control cells, averaged over an 80 kb window around each of the 24 *Asi*SI sites, correlates with the level of both SMC3 and SCC1 averaged over the same window (Figure 3C and Figure S18). This strongly suggests that the effect of cohesin on γH2AX is direct and mediated in *cis* in chromatin, rather than due to a global increase of signalling and kinase activity within the cell.

### Cohesin maintains low level of γH2AX at TSS

**Figure 4:**
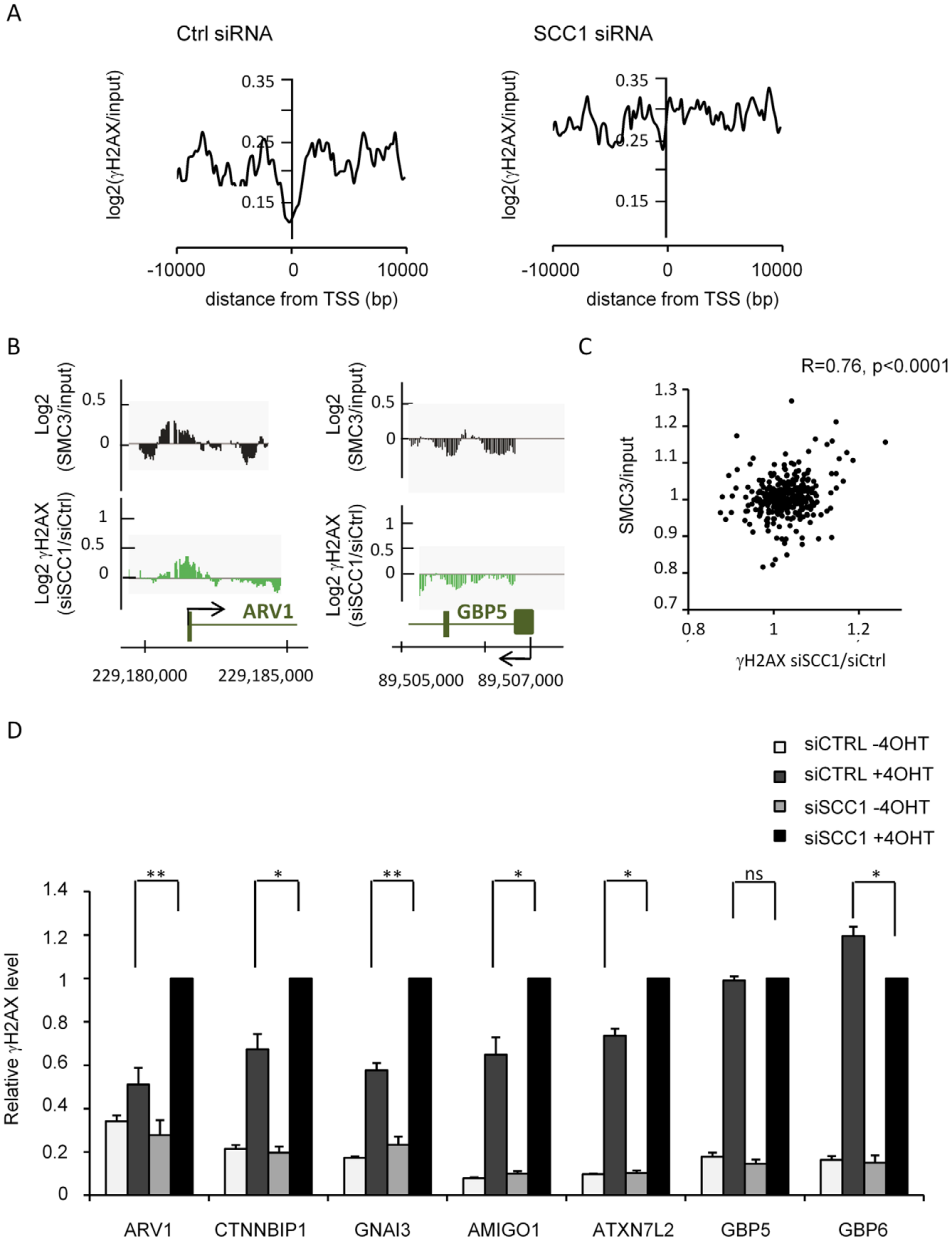
γH2AX accumulates on cohesin-bound genes and promoters in SCC-deficient cells. A, The γH2AX/input signal after 4OHT treatment (average from duplicate experiments), smoothed using a 200 bp window, is plotted relative to the TSS from all 359 genes located within γH2AX domains. The left panel shows the distribution in control cells and the right panel shows the distribution in cells transfected with SCC1 siRNA. B, Detailed views of the ChIP-chip signal from two experiments obtained on ARV1 (cohesin rich) and GBP5 (cohesin poor) promoters. Log2 SMC3/input (black) and the ratio of γH2AX in SCC1 siRNA versus Control siRNA transfected cells (expressed as Log2) (green) are presented. C, For each gene encompassed in γH2AX domains, the SMC3 signal was averaged and plotted against the ratio of γH2AX in SCC1/CTRL siRNA transfected cells. D, Levels of γH2AX at various locations were analysed by ChIP in siRNA transfected *Asi*SI-ER-U20S cells. For each primer pair, data are expressed relative to the γH2AX signal obtained in SCC1 transfected cells. The mean and standard deviation of the mean (SDOM) from four independent experiments are shown. *p* value between CTRL and SCC1 siRNA transfected cells, treated with 4OHT, are indicated above (* p<0.05, ** p<0.01).

We next examined in more detail the behaviour of γH2AX in SCC1 depleted cells, more specifically on the genes contained within γH2AX domains. We reported previously a decrease in γH2AX signal at Transcriptional Start Sites (TSS) within γH2AX domains [10]. This decrease was practically undetectable in SCC1-deficient cells, when compared to siRNA control cells (Figure 4A). Accordingly, in cells transfected with SCC1 siRNA we could observe a significant increase of γH2AX at promoters compared to control cells, whereas this increase was much less pronounced upstream or downstream TSS (Figure S19). This indicates that SCC1 depletion triggers an abnormal accumulation of γH2AX at TSS. We observed that this behaviour preferentially affects genes normally bound by cohesin (Figure 4B). For each of the 359 genes embedded in γH2AX domains, we calculated the SMC3 signal and the ratio of γH2AX in siRNA SCC1/siRNA CTRL transfected cells. When plotted against each other we could see a significant correlation (Figure 4C). The same was true when SCC1 signal was plotted (Figure S20). Along the same line, genes on which γH2AX increased the most after SCC1 depletion, significantly showed more SMC3 (upper panel) and SCC1 (lower panel) (Figure S21). This strongly suggests that the presence of cohesin prevents γH2AX spreading on genes.

We confirmed these data by Q-PCR on selected SMC3-bound (ARV1, CTNNBIP1, GNAI3, ATXN7L2, and AMIGO1) and two SMC3-unbound (GBP5 and GBP6) genes (Figure S22). Transfection with SCC1 siRNA increased γH2AX levels up to twofold on the SMC3-bound genes but did not affect the SMC3-unbound regions (Figure 4D). Altogether our data indicate that cohesin directly controls the accumulation of γH2AX on chromatin and at promoters.

### Cohesin participates in transcriptional maintenance within γH2AX domains

**Figure 5:**
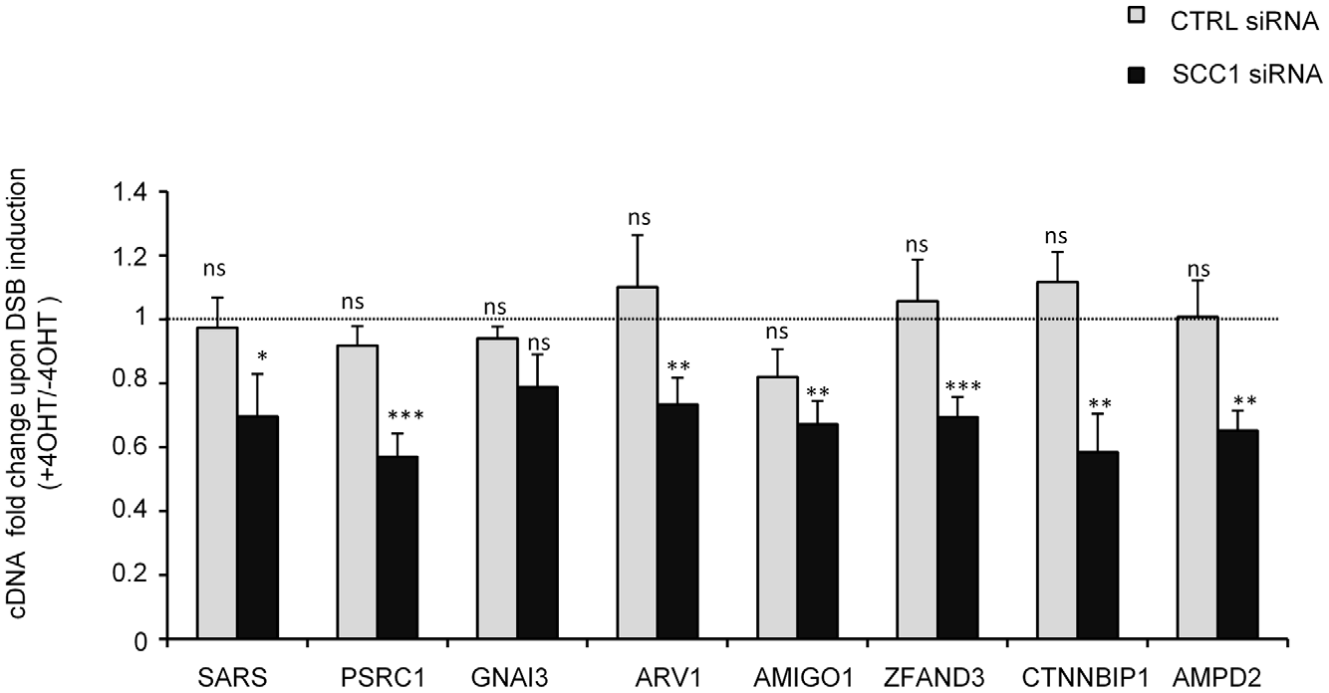
Cohesin is required to ensure transcriptional maintenance in γH2AX domains after 4OHT treatment. RNAs were prepared and reverse transcribed from *Asi*SI-ER-U20S cells transfected with control or SCC1 siRNA before and after 4 hours of 4OHT treatment (as indicated). cDNA levels for several genes encompassed in γH2AX domains were measured by Quantitative PCR. Data were normalized against P0 (ribosomal protein) cDNA. Data were expressed as cDNA fold change between 4OHT treated and untreated cells. The mean and SDOM from five independent experiments are shown. The *p* value above each bar indicates one sample t-test against a theoretical ratio of 1 (no change between +4OHT and −4OHT) (* p<0.1, ** p<0.05, *** p<0.01).

Gene transcription remains unaffected within *Asi*SI-induced γH2AX domains and active genes harbour low levels of γH2AX [10]. Since cohesin depletion led to an increase in γH2AX at cohesin-bound genes, we wondered whether transcription was still maintained after DSB induction in this cohesin-deficient context. We performed RT-QPCR for eight genes located within γH2AX domains, before and after break induction in control and SCC1 depleted cells. As expected, since cohesin plays a role in transcriptional regulation, SCC1 depletion affected the transcription of some of the tested genes, without DSB induction (Figure S23). As previously reported [10], 4OHT treatment did not alter gene expression in SCC1-proficient cells (CTRL siRNA). In contrast, gene expression decreased after 4OHT treatment in an SCC1 depleted background (Figure 5), indicating that cohesin helps to ensure normal gene expression in γH2AX domains after DSB induction.

### Cohesin and γH2AX domain boundaries

**Figure 6:**
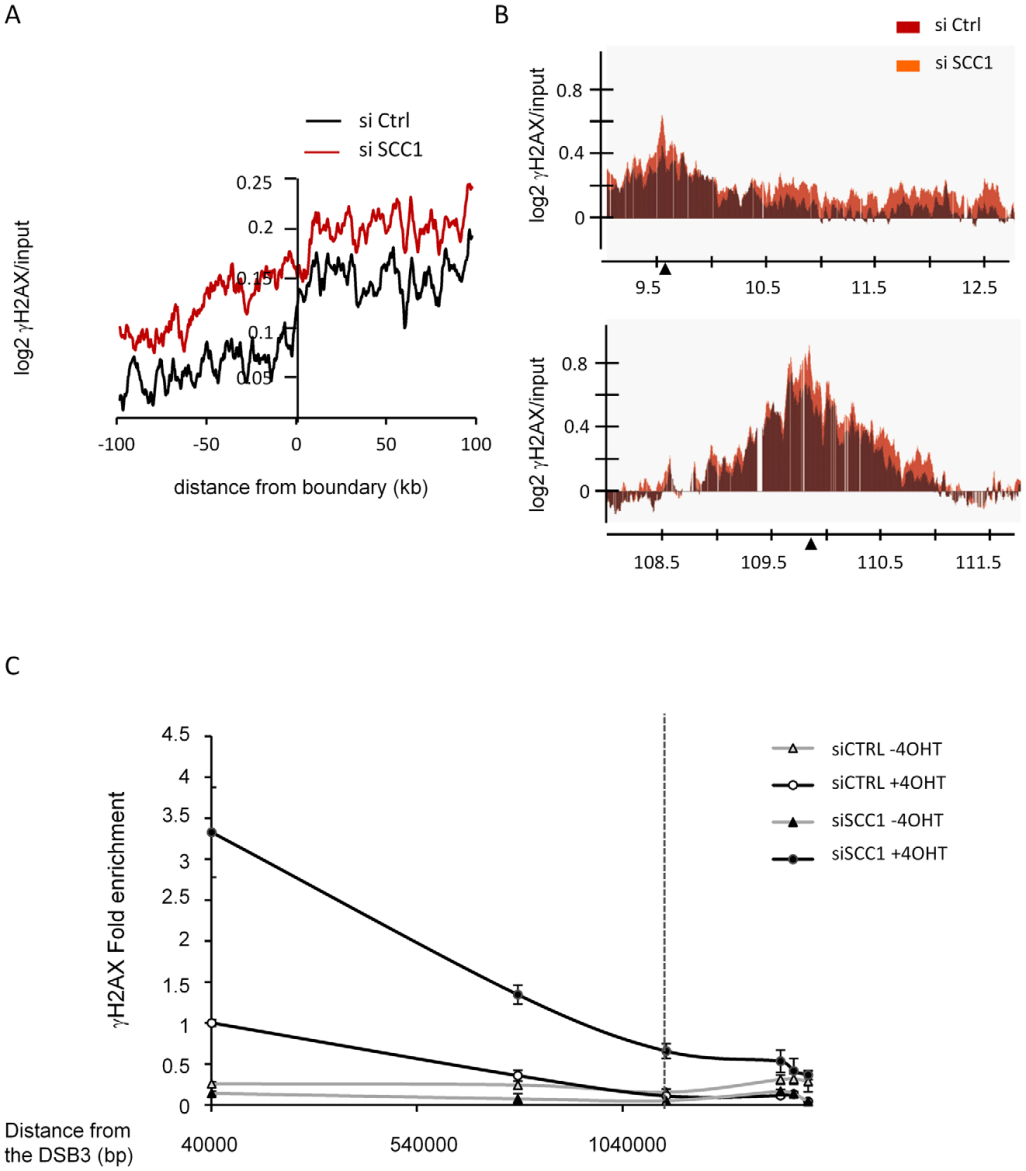
Involvement of cohesin at γH2AX domains boundaries. A, Boundaries of γH2AX domains (from the average of two 4OHT-induced γH2AX/input ChIP-chips) were determined in control cells using our previously described algorithm (see Table S2). Data were aligned and overlaid with the right and mirror left borders combined. Data are shown over a 200 kb window centered on boundaries and averaged using a 10 kb window size. Profiles are shown for γH2AX in control cells (black) and in SCC1 siRNA transfected cells (red). B, Detailed views of two γH2AX domains. The γH2AX profile from control cells (red) is presented with the γH2AX profile from SCC1 depleted cells (orange). Upper panel, the γH2AX signal, is able to spread further in SCC1 depleted cells. Lower panel, the γH2AX signal upon SCC1 depletion does not spread beyond the domain defined in control cells. Note that although both domains show different behaviors for γH2AX at the boundary, they both show a similar increase in γH2AX levels upon SCC1 depletion. C, γH2AX ChIP performed in 4OHT-treated control and SCC1 depleted cells were analyzed by Q-PCR, using primer pair located at various positions from the right boundary of the domain presented Figure 6B top panel. Data are expressed relative to the value obtained with the first primers pair (41 kb) in CTRL cells treated with 4OHT (set to 1). A representative experiment is shown. The position of the boundary identified in control cells by analyzing ChIP-chip with our algorithm is represented by a dotted line.

Finally, we examined the behaviour of γH2AX upon SCC1 depletion at γH2AX domain boundaries. Cohesin has been proposed to mediate long range interactions and to play a role in chromosome looping and 3-dimensional organisation. Thus, it appears as an intriguing candidate for restraining γH2AX spreading within defined chromosomal domains. Using γH2AX domain boundaries identified in control transfected cells (Table S2), we observed a wider spreading of γH2AX in SCC1 depleted cells than in control cells (Figure 6A). However, when we looked individually at each *Asi*SI-induced γH2AX domain, we found that some domains appeared to be cohesin-independent while others showed extended spreading upon SCC1 depletion. This difference was not a consequence of elevated γH2AX within domains, since among domains that incurred a similar increase in γH2AX upon SCC1 depletion, some domains showed extended spreading (Figure 6B top panel) while others did not (Figure 6B bottom panel). The extended spreading observed on this domain was further confirmed by QPCR analysis using primers pairs at various locations (Figure 6C). One possibility is that cohesins are directly involved in a subclass of domain boundaries where they act to constrain spreading. However, we could not find a correlation between cohesin distribution and boundary positions either globally (Figure S24A) or individually (Figure S24B). Thus, it is unlikely that cohesins are physically involved in defining the limits of γH2AX domains. Alternatively, the global increase of γH2AX that occurs upon SCC1 depletion could account for the extended spreading on chromatin (such as Figure 6B top panel) unless some other specific features constrain this spreading (such as on the domain Figure 6B bottom panel).

## Discussion

### Cohesin loading at DSBs

Taking advantage of our recently described inducible system to generate sequence specific DSBs at multiple positions, we have investigated the recruitment of cohesin at DSBs in human cells. Consistent with previous reports [21]–[24], we observed an increase of several cohesin subunits at break sites. As in yeast this recruitment likely depends on H2AX phosphorylation, since significant decrease in SMC3 and SCC1 targeting was observed when using an ATM inhibitor ([20] and our unpublished data). However, we found that cohesin recruitment was very moderate and restricted to the immediate vicinity of the DSB which is in stark contrast to the 50-kb wide cohesin loading that occurs in yeast around HO-induced DSBs [17]–[19]. Importantly, since in our system doing ChIP after 4H of 4OHT treatment allows studying all recruitment events that occur at a DSB between 0H and 4H of repair (as once in the nucleus the enzyme cuts and re-cuts the site), this difference is unlikely to be due to a difference in the kinetics of cohesin recruitment at DSBs. We also performed cohesin ChIP at 14H post-break induction, in order to make sure that in human cells cohesin targeting does not occurs at very late time point (Figure S4). In addition, we also controlled that such a restricted cohesin recruitment was not due to the high amount of DSBs induced in our cell line. We showed that soluble cohesins were not limiting after DSB induction and that a similar cohesin recruitment pattern was also observed in an I-*Sce*I cell line (single cut) (Figure S7). Thus, altogether, our data show that cohesins are only recruited to the vicinity of a DSB in human cells contrarily to the extended cohesin spreading observed in yeast.

Accumulation of cohesin around DSBs has been proposed to enhance cohesion between sister chromatids in order to promote efficient repair by homologous recombination (HR) (for review [33]). While HR accounts for the majority of repair events in yeast, DSBs are mainly repaired by Non Homologous End Joining events in mammalian cells, even during G2 phase [40]. This could thus account for the difference of cohesin spreading observed between yeast and mammalian cells. Several additional differences exist in the behaviour of cohesin complexes between yeast and metazoan. For example, yeast cohesins have been proposed to translocate along chromatin fibers, eventually accumulating at sites of convergent transcription [41]. In contrast, Drosophila and mammalian cohesins do not show any preference for convergent genes and accumulate at promoters and CTCF binding sites [34]–[36], [42]. These differences in cohesin distribution may reflect basic differences in the organization of yeast and metazoan genomes, the former being smaller and more compact, with a higher density of transcribing genes. They might also be indicative of different cohesin targeting mechanisms, which could also partake in the different localizations observed at DSBs. Finally, the absence of cohesin spreading in human cells may be compensated for by post-translational modifications that increase cohesion. Acetylation and phosphorylation of cohesin subunits at various residues are suspected to play critical roles in regulating the ATPase and translocase activity, as well as the cohesion properties of the cohesin complex (for review [15]). Thus, follow up investigations into the distribution of cohesin modifications upon DSB induction may reveal the molecular basis for the observed differences in localization between yeast and human cells. More specifically, residues 966 and 957 of SMC1, which are phosphorylated by ATM in response to damage [43]–[46], are not conserved in yeast and it is thus tempting to speculate that they could act to promote cohesion using preloaded cohesins in mammalian cells.

### Influence of cohesin on global γH2AX levels

We found that depletion of cohesin leads to a global increase of γH2AX after DSB induction (both using ChIP and immunofluorescence), in agreement with reports of γH2AX increase in irradiated, SCC1- and SMC3-depleted cells [39]. While this increase was moderate, it was reproducible and observed at several γH2AX domains (Figure S15). Our data indicate that the removal of cohesin from chromatin triggers an accumulation of γH2AX in *cis*, since this increase is found preferentially on regions normally enriched in cohesin. One hypothesis is that cohesin inhibits the establishment of H2AX phosphorylation, for example by counteracting ATM activation or/and recruitment. Alternatively, the increase in γH2AX upon SCC1 depletion could reflect impairment in the recruitment of phosphatases at breaks, such as PP2A [47]. It is interesting to note that Sugoshin, a protein that interacts with the cohesin complex and regulates cohesion in mitosis and meiosis, also interacts with PP2A [48]–[50]. One could thus envisage that cohesin recruits PP2A to chromatin and thereby regulates γH2AX levels.

### Cohesin and maintenance of transcription within γH2AX domains

Both Pol II and Pol I transcription are down regulated in the vicinity of a DSB in an ATM-dependent manner [10], [12], [13]. Whether this extinction is induced by γH2AX is not clear, since inhibition of Pol I is independant of H2AX [12] and inhibition of Pol II at least in yeast, appears to be dependent on resection rather than on γH2AX spreading [6]. We recently reported that the transcription of genes within γH2AX domains, but further away from a break, remains unchanged and that these active genes harbour reduced levels of γH2AX [10]. Here we found that this maintenance of transcription in γH2AX domains is impaired upon cohesin depletion.

First we observed a moderate but general increase of γH2AX levels on the cohesin-target genes encompassed in γH2AX domains after cohesin depletion, indicating that cohesins contribute to maintain reduced γH2AX level on genes. Secondly, for eight genes located in various domains, this was associated with a significant DSB-dependant transcriptional decrease. Since the effect of cohesin depletion on γH2AX levels occurred on most genes of the domains, it is likely that the trend observed on these eight genes is a general feature illustrating the role of cohesin in transcriptional maintenance, although further genome wide studies would be required to generalize our findings. It is also important to underline that both γH2AX increase on genes and DSB-dependant transcriptional decrease in cohesin depleted cells were quite moderate, and thus, while this could be to due siRNA efficiency, or to an asynchronous cleavage of *Asi*SI sites in the cell population, we also cannot exclude that other unrelated factor participate in the protection of active genes in γH2AX domains.

**Figure 7:**
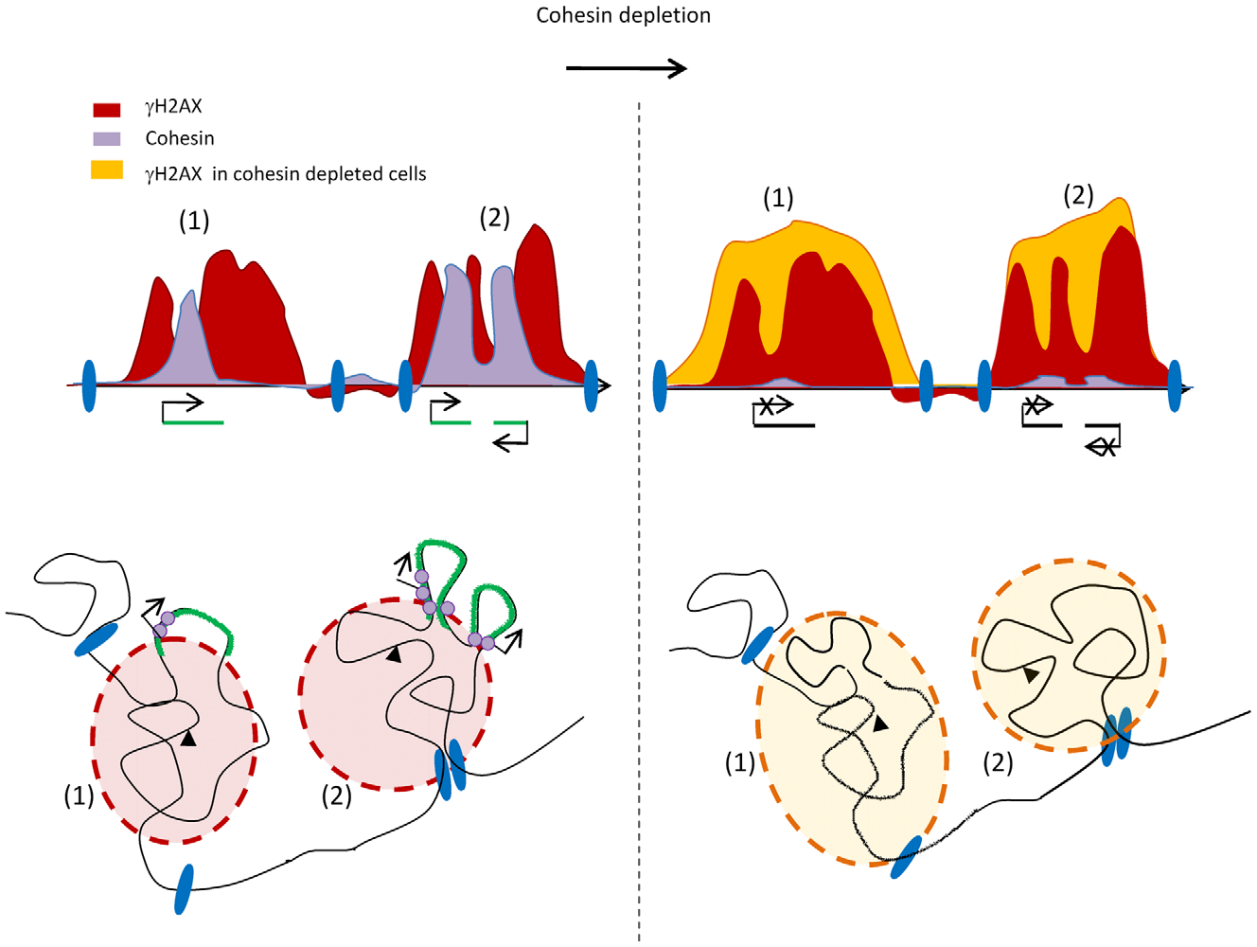
Model of 3D γH2AX spreading. Upon DSB induction (black triangle) γH2AX spreads within a nuclear space (γH2AX foci in red). Some regions within γH2AX foci could be withdrawn to be, for example transcribed (green loop), therefore leading to “holes” within the γH2AX domain, when depicted linearly (upper panel). The cohesin normally present along the chromosomes in undamaged cells, (purple circle) may play a role after DSB induction, in keeping such genes outside of γH2AX foci due to their long range interaction properties. They could thereby protect genes from the surrounding chromatin changes and ensure their correct transcription. Upon cohesin depletion (right panel) these loops would reintegrate γH2AX foci leading both to the increase of γH2AX on genes and a decrease in transcription. In addition, γH2AX foci could be demarcated by large chromosomal domains, anchored via unknown components (blue ellipses). On some domains (1), the increase in γH2AX upon cohesin depletion would extend the limits of γH2AX until it either fades away or reaches a chromosomal domain transition (blue ellipse). On other domains (2), cohesin depletion would not lead to an extension of γH2AX spreading, due to the pre-existence of a chromosomal domain transition at the boundary. These elements which define chromosomal domains transitions are unlikely to be cohesins since there is no correlation between γH2AX boundary position and cohesin binding.

This cohesin-dependant gene protection is unlikely to be a damaged–induced process since cohesin recruitment at DSB only occurs on the surrounding 2kb. Instead we favour the hypothesis that the cohesins already present on a normal, undamaged genome could protect active genes from the chromatin changes induced by DSBs, such as γH2AX which has been proposed to enhance chromatin compaction [51] and could therefore be deleterious for transcription. Interestingly, many recent studies have established a clear link between the ability of cohesin to regulate transcription and its ability to mediate chromosome looping. It is thus tempting to speculate that cohesin could protect transcription in γH2AX domains, by maintaining transcribed loci outside of γH2AX foci. This would allow to both keep low levels of γH2AX on active genes and to ensure their correct transcription post DSB induction (Figure 7).

### Cohesin, γH2AX domains boundaries, and chromosome organization

Since cohesins are known to mediate chromatin looping, they could also be involved in anchoring the chromosomal domain, within which γH2AX would spread. While we found that cohesin depletion triggers boundary expansion at some domains, we could not find a corresponding enrichment in cohesin at those positions (Figure S24). Thus, it is unlikely that cohesin plays a direct role in anchoring γH2AX domains. We believe that the global increase in γH2AX levels that occurs in the absence of cohesin, leads to extended spreading farther away from the break unless some specific constraints counteract γH2AX propagation. In order to get insights into the nature of these potential constraints, we have compared our data with the recently published Hi-C mapping of long range chromosomal interactions [52], which identified the positions of chromosomal domains, amongst other features. Remarkably, a significant proportion of γH2AX domain boundaries correlated with chromosomal domain transitions (Figures S25 and S26). In conclusion, we believe that γH2AX spreads around DSBs until it naturally fades away or it encounters a chromosomal domain transition. Fading is likely dependent on factors such as the distance from the break and the intensity of γH2AX induction, and thus cohesin depletion would trigger extended γH2AX spreading due to higher levels of γH2AX. In contrast, chromosomal domain transition stops propagation regardless of γH2AX levels, and it is unlikely that cohesins are involved in these domain transitions, since these boundaries were intact upon SCC1 depletion (not shown).

In summary, our results suggest that phosphorylation of H2AX after DSB is established on a pre-existing chromatin/chromosomal organization (Figure 7). While further investigations are required to validate such a hypothesis, it is interesting to point out that if true, γH2AX spreading might thus be used as read-out of 3-dimensional chromosome structure.

## Materials and Methods

### Antibodies

Rabbit polyclonal antibodies against SMC3 were raised using recombinant SMC3 (αSMC3-A) or an SMC3 peptide (αSMC3-B) and have been described in [53]. They were further tested and validated in human cells in [38], and in the present manuscript (Figure S1). The rabbit polyclonal antibody against SA1 was raised using a C-terminal peptide as immunogen (CEDDSGFGMPMF) and has been validated by ChIP in mouse cells (Remeseiro et al., submitted), and in human cells (this manuscript, Figure S2). The rabbit polyclonal antibody against SA2 was made against a peptide within the C-terminal region of hSA2 “EPKRLRPEDSFMSV”, and affinity purified against the antigen. This antibody was validated against human proteins in Figure S2.

### Cell culture

*Asi*SI-ER-U20S and I-*Sce*I-ER-U20S cells were cultured in Dulbecco's modified Eagle's medium (DMEM) supplemented with antibiotics, 10% FCS (Invitrogen) and 1 µg/mL puromycin at 37°C under a humidified atmosphere with 5% CO_2_. *Asi*SI-ER-T98G cells were cultured in Minimum Essential Media (MEM) GlutaMAX, supplemented with MEM Non Essential Amino Acid (NEAA), antibiotics, and 10% FCS (Invitrogen).

Synchronization of *Asi*SI-ER-T98G cells was achieved by 72 hours of serum starvation (0% FBS). Cells were collected in G1 and G2 phase after 10H and 28H, respectively, of 20% FBS re-induction. Synchronisation of *Asi*SI-ER-U20S in G2 was achieved by an 18H R0-3306 (Calbiochem) 9 µM treatment. For siRNA transfection, 5.0×10^6^ cells were electroporated with 10 µL of 100 µM siRNA using the Cell Line Nucleofector kit V (Amaxa), according to the manufacturer instructions, and collected 48H after transfection. Sequences for siRNA are displayed Table S3. When indicated, cells were treated with 300 nM 4OHT for 4H or 14H.

### RNA and RT analyses

RNA was extracted using the RNAeasy kit (Qiagen) following manufacturer instructions. 1 µg of RNA was reverse transcribed using Im-Prom^II^ RT (Promega) with random hexamers. cDNAs were analyzed by Q-PCR using primers described in Table S3 and normalized against P0 cDNA levels.

### Fractionation and immunoblotting

Cell pellets (5.10^6^ cells) were fractionated as reported [54]. Briefly, cells were first resuspended for 15 min on ice in 200 µl of 50 mM HEPES pH 7.5, 150 mM NaCl, 1 mM EDTA supplemented with the mini protease (Roche) and phosphatase inhibitor cocktail (Sigma). Following centrifugation at 14000 rpm for 5 min, the supernatant was collected (fraction I), and pellets were incubated in 200 µl of the same buffer supplemented with 0.1% triton for 15 min at 4°C. The supernatant was collected as before (fraction II). The pellets were further extracted in 200 µL of the same buffer supplemented with 0.1 mg/mL RNAse (Abcam) for 30 min on ice. The extracts were clarified by centrifugation at 14 000 rpm for 5 min (fraction III). Pellets were next resuspended in 200 µl extraction buffer supplemented with 10 nM MnCl2 and 0.07 mg/mL DNAse1 for 30 min at room temperature Western blot were performed using Invitrogen precast gels and buffer following manufacturer instructions, and using an anti-SMC3 (αSMC3 A), an anti-SCC1 (Ab992 rabbit), or an anti-H3 (Ab1791-100 rabbit).

### Chromatin immunoprecipitation

ChIP assays were carried out according to the protocol described in [55] with the following modifications. 200 µg of chromatin was immunoprecipitated using 2 µg of anti-γH2AX (Epitomics), anti-rad21 (SCC1) (Abcam ab992), anti-SMC3 (a mixture of the two rabbit homemade antibodies), anti SA1 (rabbit homemade antibody), anti SA2 (rabbit homemade antibody) or without antibody (mock). For ChIP-Q-PCR, immunoprecipitated and input DNA were analysed in triplicate by real time Q-PCR (primer sequences are provided Table S3). IP efficiency was calculated as percent of input DNA immunoprecipitated, on positive loci (such as close to a DSB) and on a negative locus (devoid of DSB). Data were expressed relative to the signal obtained on the negative locus. For ChIP-chip, DNA was amplified, labelled, and hybridized to high density oligonucleotide tiling arrays covering human chromosome 1 and 6 (Affymetrix Human Tiling 2.0R-A), using the standard Affymetrix procedure, by the GeneCore facility at EMBL Heidelberg.

### Microarray data analysis

Scanned array data were normalized using Tiling Affymetrix Software (TAS) (quantile normalization, scale set to 500) and analyzed as described in [10]. Peaks and boundaries of γH2AX domains were determined using our home made algorithm (described in [10]). Briefly, this algorithm was inspired from [56] and allows determining enriched domains of any size. Domains are determined through a two-step process. The first step defines zones of interest as contiguous sections of N probes in which x% of the probes are above a certain threshold. Second step allows bidirectional zones extension from theses seeds, to refine their limits (also based on % of probes above a certain threshold). These zones can next be merged and filtered based on their size and values. For cohesin peaks identification, we used the following settings: Contiguous sections of 20 probes with at least 17 probes above the threshold were identified (threshold was based on the percentage of graph values greater than 90% on individual chromosomes).

In order to plot data with respect to transcription start sites (TSS), gene transcript positions and orientations were obtained from the refFlat table from UCSC (hg18). All genomic coordinates were from the genome assembly NCBI Build 36.1, and annotations were retrieved from the UCSC genome browser http://genome.ucsc.edu.

Microarray probe coordinates and data have been submitted to Array Express under accession number E-TABM-1164.

### Cleavage efficiency assay

The full procedure for the cleavage assay has been previously described [10]. Briefly biotynilated double stranded oligonucleotide were ligated overnight to genomic DNA extracted from 4OHT treated or untreated *Asi*SI-ER-U20S cells. T4 ligase was heat inactivated at 65°C for 10 min, and DNA was fragmented by *Eco*RI digestion at 37°C for 2 h followed by heat inactivation at 70°C for 20 min. After a preclearing step, DNA was pulled down with streptavidin beads (Sigma) at 4°C overnight, and then washed 5 times in RIPA buffer and twice in TE. Beads were resuspended in 100 µL of water and digested with *Hind*III at 37°C for 4 h. After phenol/chloroform purification and precipitation, DNA was resuspended in 100 µL of water, and submitted to Q-PCR, using primers described in Table S3.

### Immunofluorescence and quantification

After transfection with siRNA, and 4OHT treatment for 4H, cells were fixed in PBS containing 3.7% formaldehyde for 15 min at RT, permeabilized in PBS-0.5% Triton X100 for 10 min, and blocked with 3% bovine serum albumine (BSA) for 30 min. After 2 h incubation with γH2AX antibody (Cell Signalling), cells were washed with PBS and probed for 1H with an Alexa Fluor 594 anti mouse antibody (Molecular Probes). Slides were mounted with Vectashield (Vector Laboratories), and images were acquired using a Leica microscope equipped with a charge-coupled device camera (CoolSNAP ES; Roper Industries), and the MetaMorph software (MDS Analytical Technologies).

Quantification of fluorescence levels was done on a least 100 nuclei using home-developed macros in ImageJ software (National Institutes of Health, Bethesda, MA) to normalize background, thresholds and measures.

## Supporting Information

Figure S1 Validation of SMC3 antibodies. A, The specificity of SMC3 antibodies (α-SMC3-A or α-SMC3-B as indicated) was analyzed by western blot with HeLa nuclear extracts. B, HeLa nuclear extracts were immunoprecipitated using either a α-SMC3-A or α-SMC3-B antibodies as indicated. Flowthrough and immunoprecipitated samples were analyzed by western blot probed with α-SMC3-A or anti-SCC1 (Abcam) C, ChIP analyses were performed in *Asi*SI-ER-U20S cells without 4OHT treatment, using a mix of the two SMC3 antibodies or no antibody (mock), as indicated. SMC3 enrichment was scored by Quantitative Real Time PCR (Q-PCR) on a previously characterized cohesin binding site [35] and on the *gapdh* promoter (negative control). ChIP efficiency was calculated as % of input DNA immunoprecipitated. A representative experiment is shown.
(PDF)

Figure S2 Validation of SA1/SA2 antibodies. SA1 and SA2 antibodies were validated by western blot with HeLa nuclear extract (A), using control or SA1/SA2 siRNA transfected HeLa cells extracts (B), and in ChIP assay followed by Q-PCR (C) using a previously characterized cohesin binding site as a positive control [35], and the *gapdh* promoter as a negative control. ChIP efficiency was calculated as % of input DNA immunoprecipitated. A representative experiment is shown.
(PDF)

Figure S3 Cohesin ChIP in synchronized cells. A, ChIP against SCC1 was performed in *Asi*SI-ER-U20S cells synchronized in G2 upon RO-3306 treatment (18H at 9 µM), before and after 4OHT, and analyzed by Q-PCR. Fold enrichment at two DSBs is shown relative to the negative locus (devoid of *Asi*SI sites). Note that 4OHT dependant recruitment of SCC1 at DSBs in G2 is similar to the changes observed upon DSB induction in asynchronous cells (Figure 1B). A representative experiment is shown. B, SCC1 ChIP was performed in *Asi*SI-ER-T98G cells synchronized in G1 or G2, using serum starvation. Targeting of SCC1 was scored by Q-PCR before and after 4OHT treatment. The mean and SDOM from 3 independent experiments of the fold enrichment observed on two DSBs relative to the negative locus is shown.
(PDF)

Figure S4 SCC1 recruitment at DSBs does not increase over time. *Asi*SI-ER-U20S cells, either untreated or treated with 4OHT during 4H or 14H, were subjected to ChIP analyses using SCC1 (Abcam) or γH2AX antibodies as indicated. Enrichment was scored by Q-PCR in the vicinity of an *Asi*SI-induced DSBs (DSB1) and normalized to the signal observed on a genomic location devoid of DSBs. A representative experiment is shown.
(PDF)

Figure S5 Profile of SMC3 in *Asi*SI-ER-U20S. A, Detailed view of the SMC3/input (black) in untreated *Asi*SI-ER-U20S cells and SCC1/input (grey) from HeLa cells, retrieved from [35]. ChIP-chip data, expressed as log2 are shown from selected areas of chromosome 1. B, The location and orientation of the 3072 genes located on chromosome 1 and 6 were used to subset data with the transcribed sequence on the right hand side. The log2 SMC3/input signal in *Asi*SI-ER-U20S cells (upper panel) and the SCC1/input signal in HeLa cells (retrieved from [35]) (lower panel) were plotted using a 200 bp sliding window for averaging and are shown over a 20 kb window centered on the TSS.
(PDF)

Figure S6 Cohesin accumulation at DSBs is statistically significant and does not occur at random positions. Three independent random simulations were run with 100 sets of sites designed to be similar in size (single points), distribution (scattered uniformly across both chr 1 and 6) and number (24 sites per random run) to the actual *Asi*SI sites. Paired t-test was performed on the average value of data points within a 2000 bp window centered on the site using SMC3 data from −4OHT and +4OHT samples, as in Figure 1F. The *p* values were logged (base 10) and box plotted to demonstrate the significance of the t-test *p* value calculated around *Asi*SI sites (Figure 1F) depicted as a red line.
(PDF)

Figure S7 The lack of cohesin spreading around DSBs is not due to a limited amount of soluble cohesin. A, Soluble (chromatin unbound) and insoluble (chromatin bound) fraction were prepared from *Asi*SI-ER U20S 4OHT-treated or untreated cells. Western blot against SMC3 and SCC1 showed that the soluble pool of cohesin is not depleted after DSB induction. B, ChIP against SCC1 was performed in I-*Sce*I-ER U20S cells before and after 4OHT treatment. The fold enrichment relative to a negative locus was scored by Q-PCR at the immediate vicinity of the I-SceI break (300 bp), and further away (2.4 kb), as indicated. A representative experiment is shown.
(PDF)

Figure S8 γH2AX is depleted at cohesin binding sites. Regions enriched in SCC1 were identified using the algorithm detailed in [10] and in the Material and Methods section (applied on SCC1 ChIP-chip data in HeLa cells [35]). Binding sites located within γH2AX domains were selected and the averaged SCC1 (top panel) and γH2AX (bottom panel) profiles around these positions are presented.
(PDF)

Figure S9 Cohesin rich genes show a low level of γH2AX. The average Log2 (γH2AX/input) (y axis) and Log2 (cohesin/input) (x axis) were calculated over the entire length of each of the 359 genes encompassed within γH2AX domains, and plotted against each other. Results are shown for SMC3 (top panel), and SCC1 retrieved from the HeLa dataset [35] (bottom panel). The correlation coefficient and the *p* value are indicated.
(PDF)

Figure S10 Profile of SCC1 in *Asi*SI-ER-U20S. A, Detailed view of the SCC1 distribution observed in HeLa cells retrieved from [35] (black, upper panel), and SCC1 observed in untreated *Asi*SI-ER-U20S cells (grey, lower panel). ChIP-chip data are presented as Log2 (Signal/input) on selected areas of the chromosome 1. B, The location and orientation of the 3072 genes located on chromosome 1 and 6 were used to subset data with the transcribed sequence on the right hand side. The Log2 (SCC1/input) obtained in *Asi*SIER-U20S cells was plotted using a 200 bp sliding window for averaging and is shown over a 20 kb window centered on the TSS.
(PDF)

Figure S11 SCC1 counteracts γH2AX. A, Detailed views of the γH2AX/H2AX signal (red) and the SCC1/input signal (black) around an *Asi*SI site (arrow), expressed as log2 and smoothed using a 500 probes sliding window. B, The average Log2 (γH2AX/input) (y axis) and Log2 (SCC1/input) (x axis) were calculated over the entire length of each of the 359 genes encompassed within γH2AX domains, and plotted against each other. The correlation coefficient and the *p* value are indicated.
(PDF)

Figure S12 SCC1 is depleted at the RNA level, protein level, and on chromatin upon SCC1 siRNA treatment. A, *Asi*SI-ER-U20S cells were transfected by electroporation with control (CTRL) or SCC1 siRNA. 48 hours after transfection, mRNA was extracted, reverse transcribed and the amounts of SCC1 and ribosomal phosphoprotein P0 cDNAs were measured by Quantitative real time PCR. SCC1 cDNA levels are shown relative to P0 levels. The mean and standard deviation of the mean (SDOM) from 3 independent experiments are shown. B, SCC1 protein level was analyzed by western blot in Control or SCC1 siRNA transfected *Asi*SI-ER-U20S cells (using Abcam SCC1 antibody ab992, upper panel). The same blot was also probed for alpha-tubulin as a loading control (lower panel). C, The depletion of chromatin-bound SCC1 by siRNA was analyzed by ChIP in siRNA transfected *Asi*SI-ER-U20S cells, using an anti SCC1antibody (Abcam ab992) or no antibody. SCC1 enrichment was assessed by Q-PCR on the four regions further described in Figure S22 (two cohesin-bound, and two cohesin-unbound regions). The mean of the relative enrichment of SCC1 over no antibody, from three independent experiments are plotted with SDOM.
(PDF)

Figure S13 Example γH2AX profiles around *Asi*SI sites upon SCC1 depletion. Detailed views, around selected *Asi*SI sites, indicated by arrows (upper and middle panels) and on two genomic regions devoid of *Asi*SI sites (lower panels). γH2AX enrichment over input in control (dark red) and in siRNA SCC1 (light red) transfected *Asi*SI-ER-U20S cells, are shown expressed as log2 and smoothed using a 500 probes sliding window. ChIP-chip analysis was performed using chromatin from *Asi*SI-ER-U20S cells treated with 4OHT. The average of two independent experiments is shown. Note that within domains, the γH2AX signal increases upon SCC1 depletion. Genomic coordinates (x-axis) are indicated in megabase (MB).
(PDF)

Figure S14 γH2AX increases around DSBs in SCC1 depleted cells. A, The average Log2 (γH2AX/input) was calculated over a 4 kb window (left panel) or an 80 kb window (right panel) surrounding *Asi*SI sites, in cells transfected with control or SCC1 siRNA as indicated. The box plots represent the distribution of the values obtained for the 24 *Asi*SI sites. The γH2AX level in SCC1 depleted cells is significantly different from the level observed in control cells. B, Same as in A except that random windows of 4 kb (left panel), and 80 kb (right panel) outside γH2AX domains were averaged.
(PDF)

Figure S15 4OHT-induced γH2AX increases in SCC1 depleted cells compared to control cells. *Asi*SI-ER-U20S cells were transfected with Control (CTRL) or SCC1 siRNA for 48 hours. Untreated or 4OHT treated cells were subjected to ChIP analyses against γH2AX. Enrichment was scored by Q-PCR within 5 γH2AX domains. Distances of the primers from the DSB are indicated. Data are normalized to the signal observed on a genomic location devoid of DSBs. Representative experiments are shown.
(PDF)

Figure S16 SCC1 depletion does not change the cleavage efficiency of *Asi*SI sites. Genomic DNA was extracted from siRNA transfected *Asi*SI-ER-U20S cells treated or not with 4OHT for 4H and assayed for cleavage at *Asi*SI sites. Pulled down DNA was analyzed by quantitative PCR amplification using primers close to two cleaved *Asi*SI sites. The mean and standard deviation of the mean (SDOM) from 3 independent experiments are shown.
(PDF)

Figure S17 SCC1 depletion leads to an increase of γH2AX as detected by immunofluorescence. *Asi*SI-ER-U2OS cells transfected with Control (CTRL) or SCC1 siRNA for 48H were treated with 4OHT for 4H and subjected to γH2AX immunofluorescence (Cell Signaling). Images were quantified and classified based on the percentage of their nucleus covered by γH2AX staining. Data are represented as the percentage of cells falling in each of four different categories. The mean and SDOM of three independent experiments are shown. (* p<0.05; ** p<0.01)
(PDF)

Figure S18 γH2AX increases on SCC1 rich domains upon SCC1 depletion. The averaged SCC1 signal over an 80 kb window around each *Asi*SI site was calculated (x-axis) and plotted against the γH2AX ratio in cells transfected with siRNA SCC1 versus siRNA CTRL(y axis). Pearson correlation and *p* value are indicated.
(PDF)

Figure S19 γH2AX increases at TSS upon SCC1 depletion. The average Log2 (γH2AX/input) upstream to promoters (−2000 to −1600 bp) (left panel), at promoters (−200 to +200 bp) (middle panel) and downstream of promoters (+1600 to −2000 bp) (right panel) for the 359 genes encompassed in γH2AX domains were calculated, in Control and SCC1 siRNA transfected cells as indicated. Distributions are represented as box plots. The *p* values (paired t-test) are indicated above. Note that the biggest increase of γH2AX upon SCC1 depletion occurs at TSSs. While we can see a significant increase both upstream and downstream, it is much weaker than the increase observed at the TSS.
(PDF)

Figure S20 γH2AX increases on SCC1 rich genes upon SCC1 depletion. For each gene encompassed in γH2AX domains, the SCC1 signal was averaged and plotted against the ratio of γH2AX in SCC1 depleted versus control cells. Pearson correlation and *p* value are indicated.
(PDF)

Figure S21 Genes showing high changes in γH2AX between SCC1 siRNA and control cells, show higher levels of cohesin. As for Figure S19, for each gene encompassed in γH2AX domains, the ratio of γH2AX in SCC1 depleted versus control cells and the SMC3 signal (top panel) were averaged. The box plots show the difference in SMC3 (top panel) or SCC1 (bottom panel) between genes showing low (<0.95) and high (>1.1) γH2AX (SCC1/CTRL) ratio. The genes on which γH2AX increases the most after SCC1 depletion, show significantly more SMC3 (upper panel) and SCC1 (lower panel).
(PDF)

Figure S22 Detailed views of the areas analyzed by Q-PCR with SCC1 siRNA. Detailed views of the SMC3/input (black) and γH2AX/H2AX (red) ChIP-chip data, on five cohesin-bound regions (A–E) and two cohesin-unbound regions (F–G). Positions of the primer pairs used for the Q-PCR analysis presented in Figure 4 are shown (arrows and grey boxes), as well as the position and orientation of genes.
(PDF)

Figure S23 Expression fold changes upon 4OHT treatment and SCC1 depletion. *Asi*SI-ER-U20S cells were transfected with the indicated siRNAs. After 48 h, cells were treated or not with 4OHT as indicated. Total RNAs were extracted and reverse transcribed. The amount of each cDNA was measured by quantitative real-time PCR, divided by the amount of P0 cDNA and calculated relative to 1 for cells transfected with the control siRNAs and not treated with 4OHT.
(PDF)

Figure S24 Cohesin binding does not correlate with boundary position. A, Boundaries of γH2AX domains were aligned and overlaid (right and mirror left borders are combined). Data are shown over a 200 kb window centered on domain boundaries and averaged using a 10 kb window size. Profiles are shown for γH2AX (red) and for SMC3 (black) in control cells. Note that we cannot see a specific increase of SMC3 at the boundary. B, Detailed views of right boundaries from two γH2AX domains. Signals for SMC3 (black), γH2AX in control cells (red) and γH2AX in SCC1 depleted cells (orange) are presented. The upper domain shows an extension of γH2AX upon SCC1 depletion, while the lower domain does not (grey arrows). However this is not correlated with the presence of SMC3 at the boundaries (black).
(PDF)

Figure S25 Comparison of γH2AX boundaries with chromosomal domain transitions. Hi-C realized with the human lymphoblastoid cell line GM06990, led to the identification of chromosomal domains at various scales, from the nucleus scale (such as the open and closed chromatin compartments) to a megabase scale. Chromosomal domains could be easily visualized using a heatmap to depict intrachromosomal interactions [52], [57]. The spatial compartmentalization is illustrated by the squared motifs on the heatmap and thus a transition between chromatin domains appears as a “node” between the squares. This level of chromosomal organization only marginally differs between cell lines [52] thus we compared the Hi-C data with our γH2AX profiling data. Inspection of various γH2AX domains, using this representation of loci interaction, showed a clear correlation between our γH2AX domains boundaries in *Asi*SI-ER-U20S cells and the spatial compartmentalization observed in GM06990 cells (see arrows).
(PDF)

Figure S26 Averaged interaction matrix around γH2AX domain boundaries. The Hi-C interaction matrix [52] located from −1 MB to +1 MB around each identified γH2AX domain boundary (23 *Asi*SI domains i.e. 46 boundaries) were retrieved and averaged (left and mirror right boundaries were combined). The averaged boundary (the 0 position) correlates with a chromosomal domain transition found using Hi-C.
(PDF)

Table S1 List of cleaved *Asi*SI sites on chromosome 1 and chromosome 6. All genomic coordinates are from the genome assembly NCBI Build 36.1. The *Asi*SI sites efficiently cleaved were determined thanks to our previous analysis using both the γH2AX signal and the cleavage signal [10].
(PDF)

Table S2 List of γH2AX domain boundaries on chromosome 1 and chromosome 6 determined using the γH2AX signal in siRNA control transfected cells. All genomic coordinates are from the genome assembly NCBI Build 36.1. The boundaries positions were determined using the algorithm described in [10].
(PDF)

Table S3 List of primers used in this study.
(PDF)
