# Supplementary figures and images for: Cohesin Protects Genes against γH2AX Induced by DNA Double-Strand Breaks
Source: PLoS Genet. 2012 Jan 19;8(1):e1002460. doi: 10.1371/journal.pgen.1002460 (PMC3261922; doi:10.1371/journal.pgen.1002460)

A

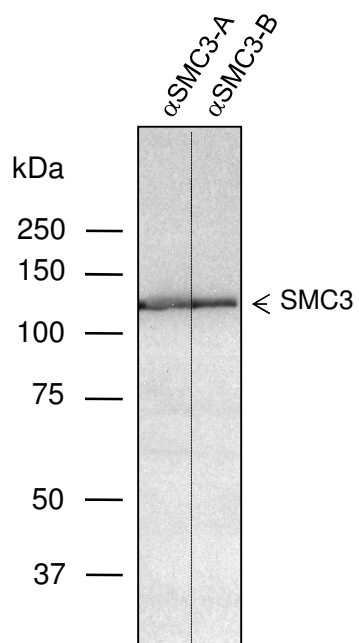

B

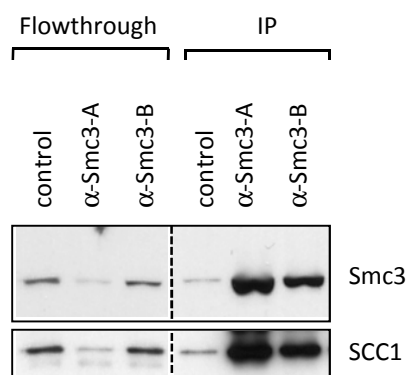

C

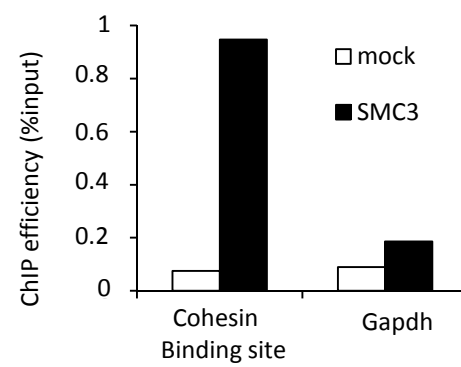

Supplement: Figure S1 — Validation of SMC3 antibodies. A, The specificity of SMC3 antibodies (α-SMC3-A or α-SMC3-B as indicated) was analyzed by western blot with HeLa nuclear extracts. B, HeLa nuclear extracts were immunoprecipitated using either a α-SMC3-A or α-SMC3-B antibodies as indicated. Flowthrough and immunoprecipitated samples were analyzed by western blot probed with α-SMC3-A or anti-SCC1 (Abcam) C, ChIP analyses were performed in AsiSI-ER-U20S cells without 4OHT treatment, using a mix of the two SMC3 antibodies or no antibody (mock), as indicated. SMC3 enrichment was scored by Quantitative Real Time PCR (Q-PCR) on a previously characterized cohesin binding site [35] and on the gapdh promoter (negative control). ChIP efficiency was calculated as % of input DNA immunoprecipitated. A representative experiment is shown. (PDF) [file pgen.1002460.s001.pdf]

A

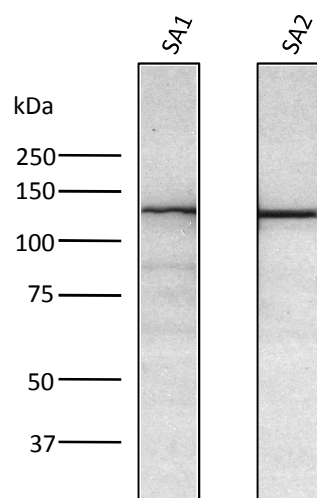

B

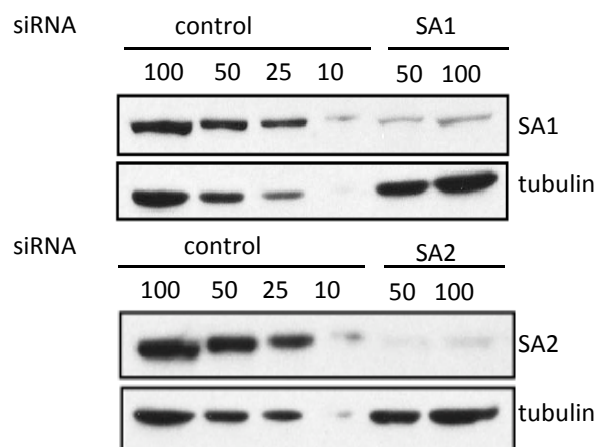

C

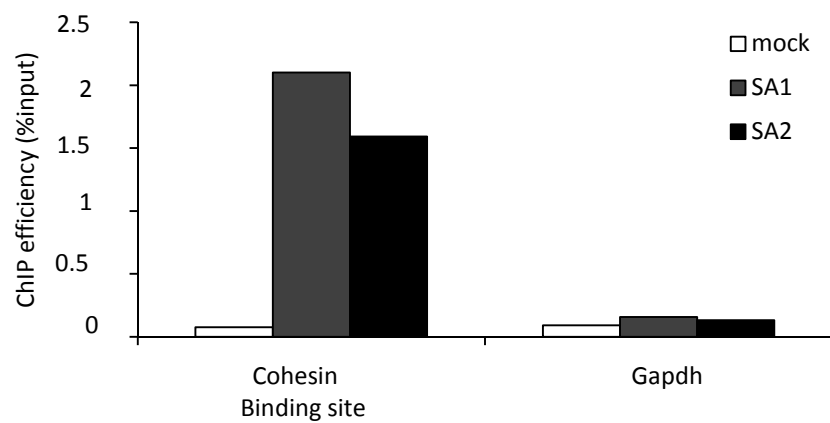

Supplement: Figure S2 — Validation of SA1/SA2 antibodies. SA1 and SA2 antibodies were validated by western blot with HeLa nuclear extract (A), using control or SA1/SA2 siRNA transfected HeLa cells extracts (B), and in ChIP assay followed by Q-PCR (C) using a previously characterized cohesin binding site as a positive control [35], and the gapdh promoter as a negative control. ChIP efficiency was calculated as % of input DNA immunoprecipitated. A representative experiment is shown. (PDF) [file pgen.1002460.s002.pdf]

A

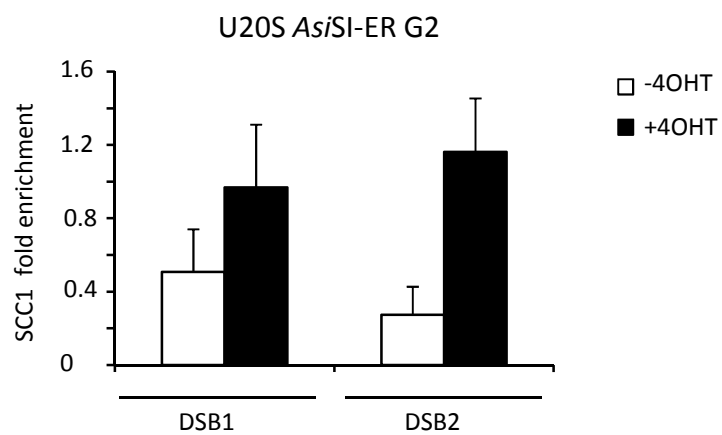

B

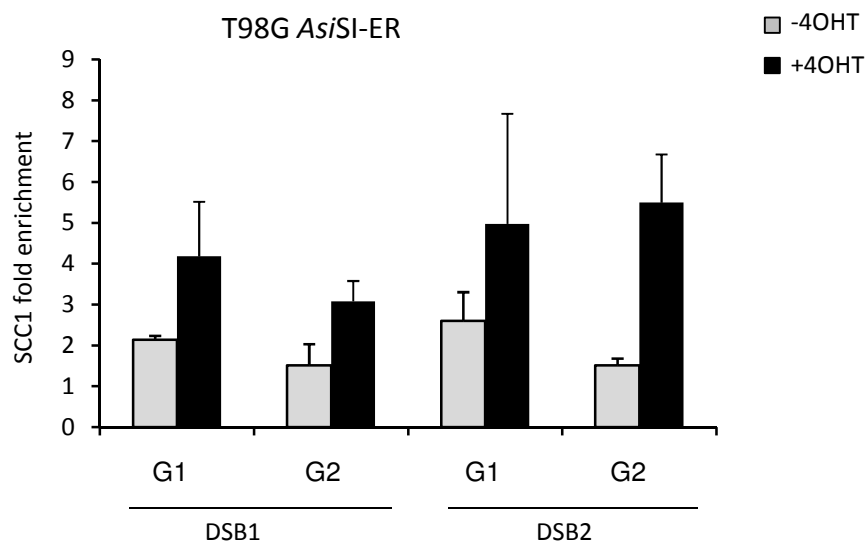

Supplement: Figure S3 — Cohesin ChIP in synchronized cells. A, ChIP against SCC1 was performed in AsiSI-ER-U20S cells synchronized in G2 upon RO-3306 treatment (18H at 9 µM), before and after 4OHT, and analyzed by Q-PCR. Fold enrichment at two DSBs is shown relative to the negative locus (devoid of AsiSI sites). Note that 4OHT dependant recruitment of SCC1 at DSBs in G2 is similar to the changes observed upon DSB induction in asynchronous cells (Figure 1B). A representative experiment is shown. B, SCC1 ChIP was performed in AsiSI-ER-T98G cells synchronized in G1 or G2, using serum starvation. Targeting of SCC1 was scored by Q-PCR before and after 4OHT treatment. The mean and SDOM from 3 independent experiments of the fold enrichment observed on two DSBs relative to the negative locus is shown. (PDF) [file pgen.1002460.s003.pdf]

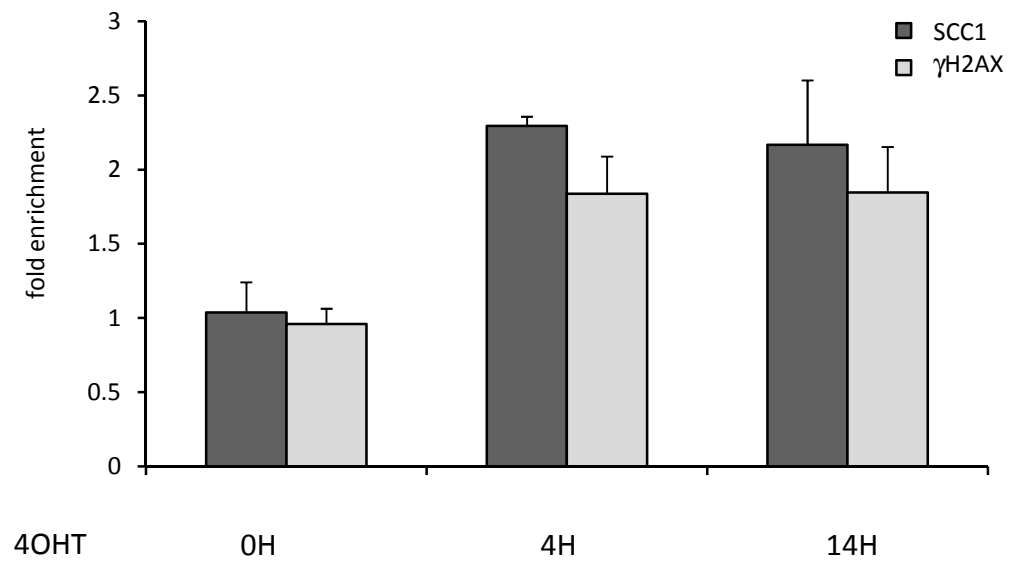

Supplement: Figure S4 — SCC1 recruitment at DSBs does not increase over time. AsiSI-ER-U20S cells, either untreated or treated with 4OHT during 4H or 14H, were subjected to ChIP analyses using SCC1 (Abcam) or γH2AX antibodies as indicated. Enrichment was scored by Q-PCR in the vicinity of an AsiSI-induced DSBs (DSB1) and normalized to the signal observed on a genomic location devoid of DSBs. A representative experiment is shown. (PDF) [file pgen.1002460.s004.pdf]

A

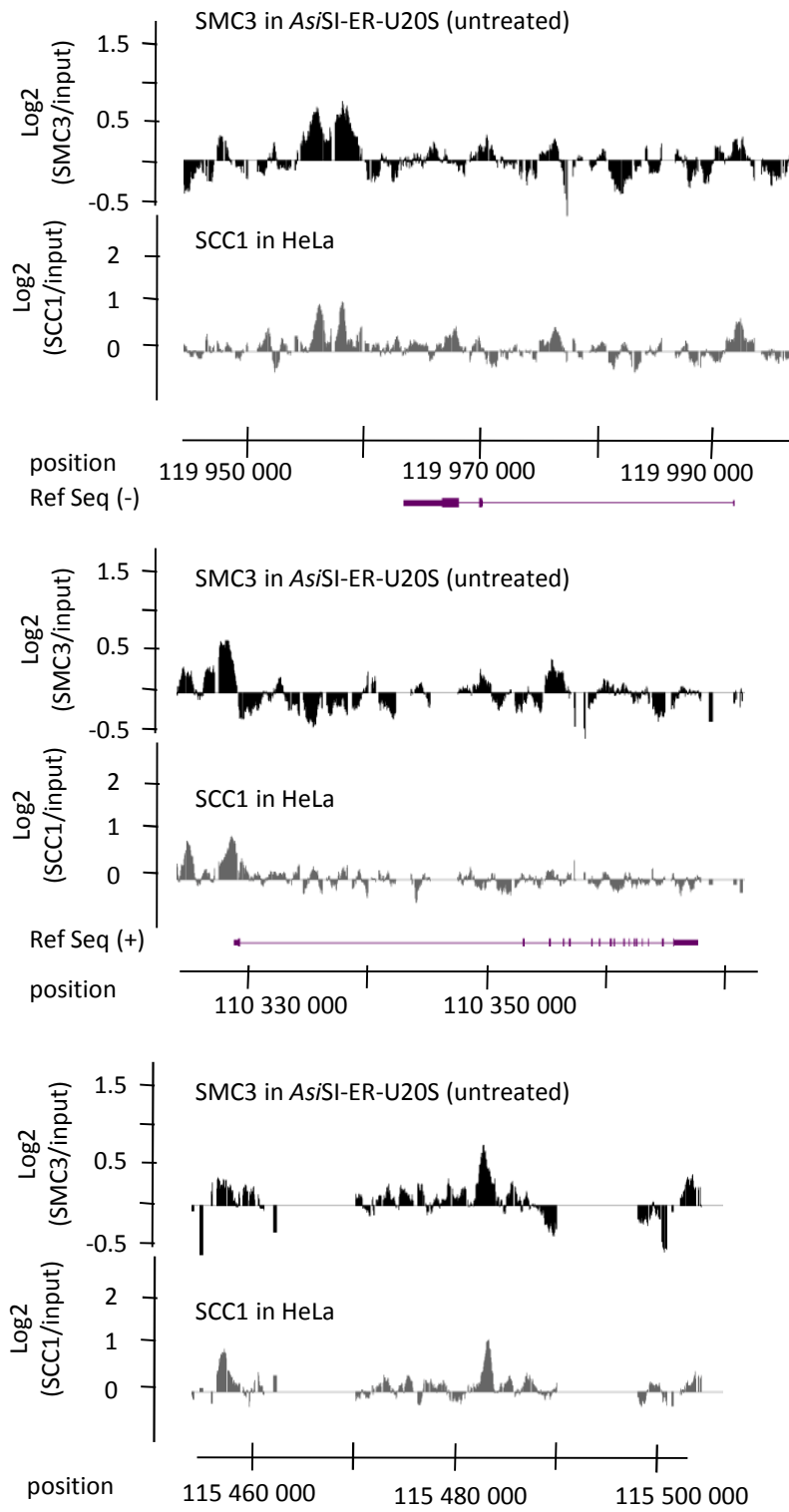

B

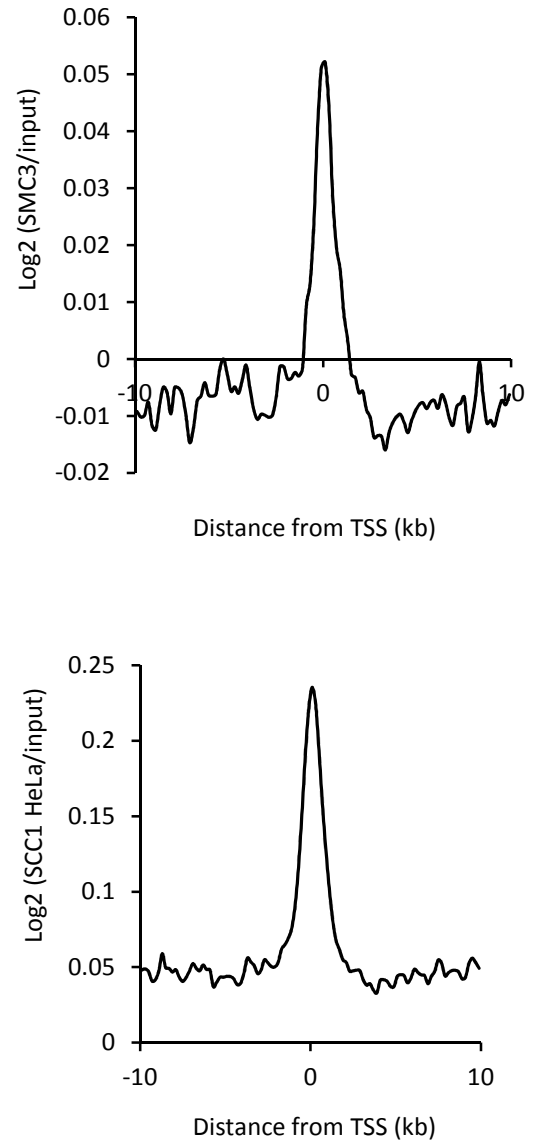

Supplement: Figure S5 — Profile of SMC3 in AsiSI-ER-U20S. A, Detailed view of the SMC3/input (black) in untreated AsiSI-ER-U20S cells and SCC1/input (grey) from HeLa cells, retrieved from [35]. ChIP-chip data, expressed as log2 are shown from selected areas of chromosome 1. B, The location and orientation of the 3072 genes located on chromosome 1 and 6 were used to subset data with the transcribed sequence on the right hand side. The log2 SMC3/input signal in AsiSI-ER-U20S cells (upper panel) and the SCC1/input signal in HeLa cells (retrieved from [35]) (lower panel) were plotted using a 200 bp sliding window for averaging and are shown over a 20 kb window centered on the TSS. (PDF) [file pgen.1002460.s005.pdf]

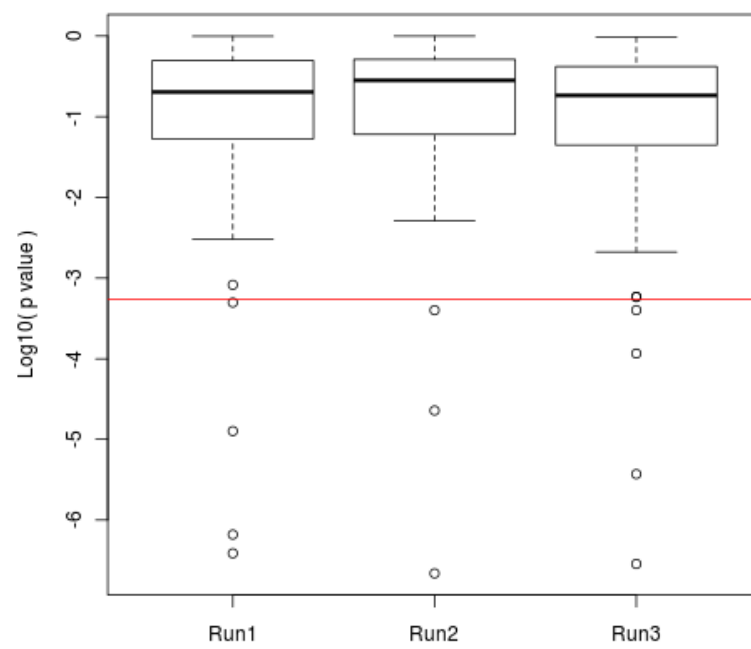

Supplement: Figure S6 — Cohesin accumulation at DSBs is statistically significant and does not occur at random positions. Three independent random simulations were run with 100 sets of sites designed to be similar in size (single points), distribution (scattered uniformly across both chr 1 and 6) and number (24 sites per random run) to the actual AsiSI sites. Paired t-test was performed on the average value of data points within a 2000 bp window centered on the site using SMC3 data from −4OHT and +4OHT samples, as in Figure 1F. The p values were logged (base 10) and box plotted to demonstrate the significance of the t-test p value calculated around AsiSI sites (Figure 1F) depicted as a red line. (PDF) [file pgen.1002460.s006.pdf]

A

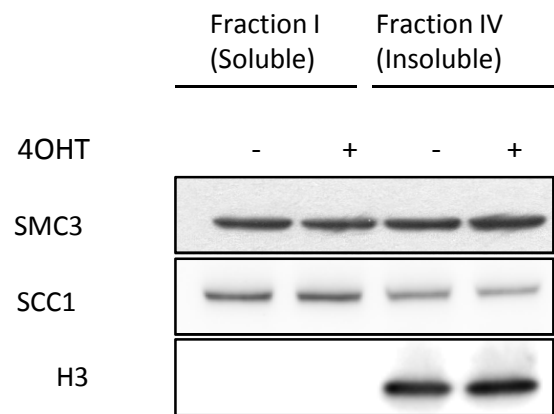

B

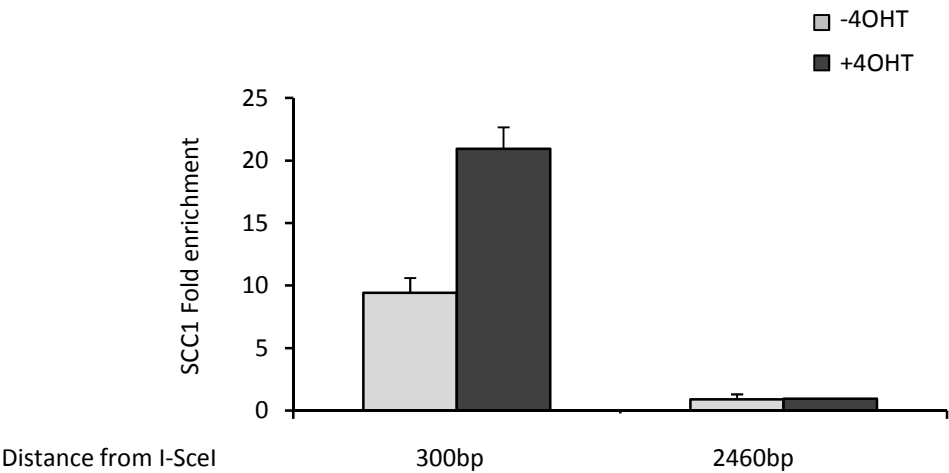

Supplement: Figure S7 — The lack of cohesin spreading around DSBs is not due to a limited amount of soluble cohesin. A, Soluble (chromatin unbound) and insoluble (chromatin bound) fraction were prepared from AsiSI-ER U20S 4OHT-treated or untreated cells. Western blot against SMC3 and SCC1 showed that the soluble pool of cohesin is not depleted after DSB induction. B, ChIP against SCC1 was performed in I-SceI-ER U20S cells before and after 4OHT treatment. The fold enrichment relative to a negative locus was scored by Q-PCR at the immediate vicinity of the I-SceI break (300 bp), and further away (2.4 kb), as indicated. A representative experiment is shown. (PDF) [file pgen.1002460.s007.pdf]

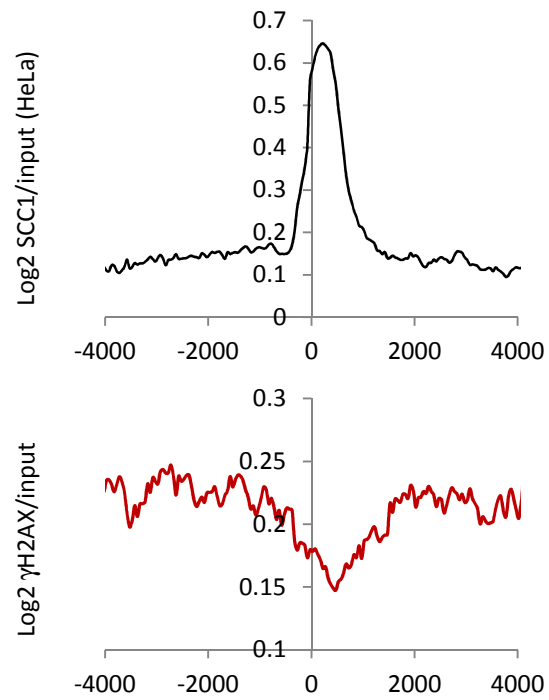

Supplement: Figure S8 — γH2AX is depleted at cohesin binding sites. Regions enriched in SCC1 were identified using the algorithm detailed in [10] and in the Material and Methods section (applied on SCC1 ChIP-chip data in HeLa cells [35]). Binding sites located within γH2AX domains were selected and the averaged SCC1 (top panel) and γH2AX (bottom panel) profiles around these positions are presented. (PDF) [file pgen.1002460.s008.pdf]

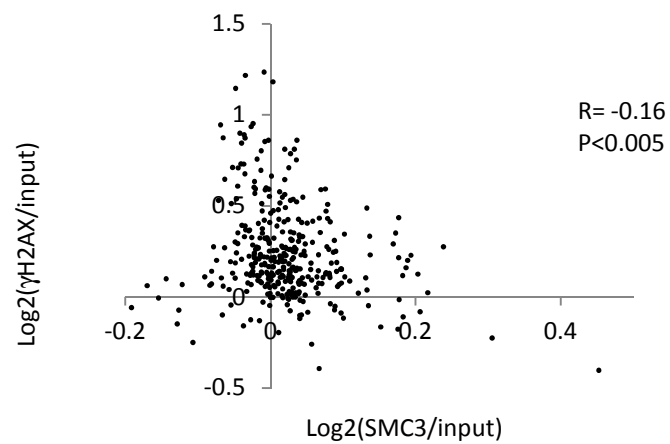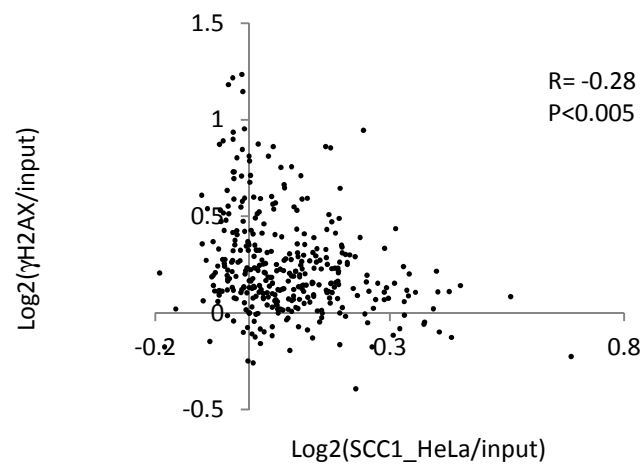

Supplement: Figure S9 — Cohesin rich genes show a low level of γH2AX. The average Log2 (γH2AX/input) (y axis) and Log2 (cohesin/input) (x axis) were calculated over the entire length of each of the 359 genes encompassed within γH2AX domains, and plotted against each other. Results are shown for SMC3 (top panel), and SCC1 retrieved from the HeLa dataset [35] (bottom panel). The correlation coefficient and the p value are indicated. (PDF) [file pgen.1002460.s009.pdf]

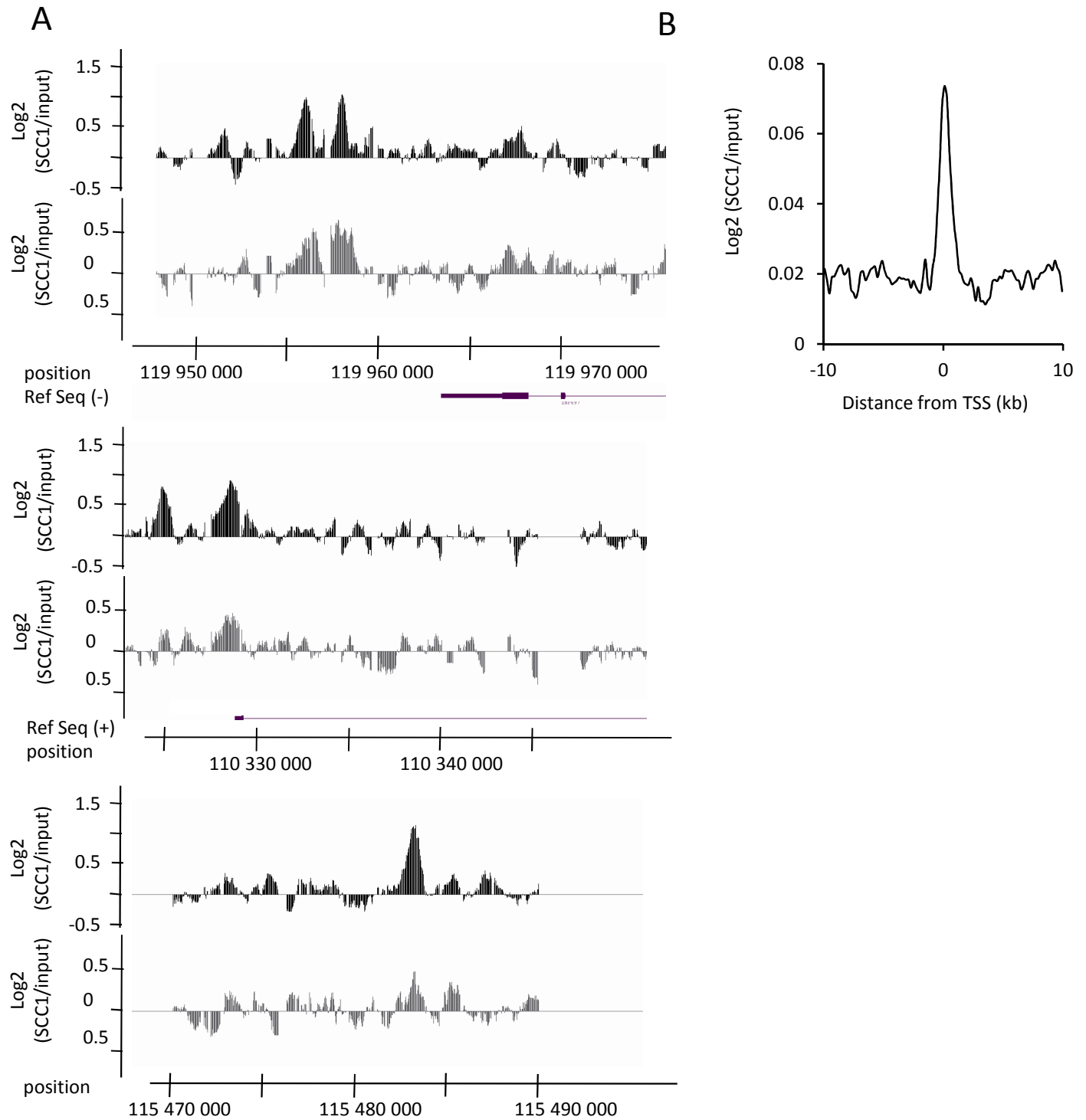

Supplement: Figure S10 — Profile of SCC1 in AsiSI-ER-U20S. A, Detailed view of the SCC1 distribution observed in HeLa cells retrieved from [35] (black, upper panel), and SCC1 observed in untreated AsiSI-ER-U20S cells (grey, lower panel). ChIP-chip data are presented as Log2 (Signal/input) on selected areas of the chromosome 1. B, The location and orientation of the 3072 genes located on chromosome 1 and 6 were used to subset data with the transcribed sequence on the right hand side. The Log2 (SCC1/input) obtained in AsiSIER-U20S cells was plotted using a 200 bp sliding window for averaging and is shown over a 20 kb window centered on the TSS. (PDF) [file pgen.1002460.s010.pdf]

A

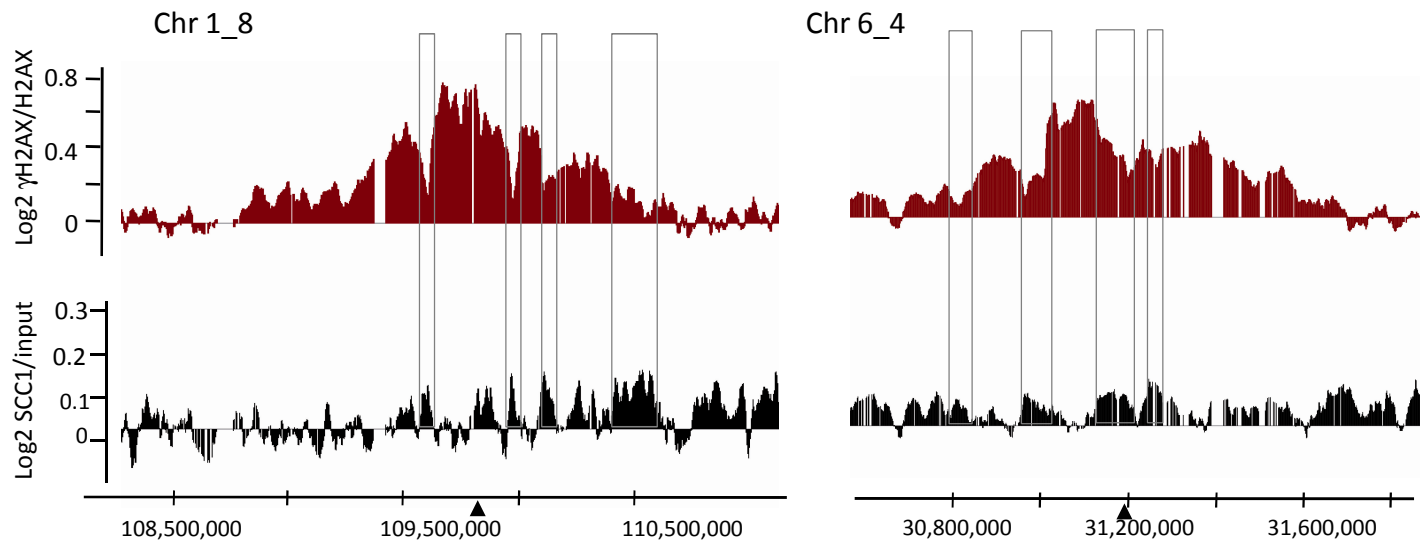

B

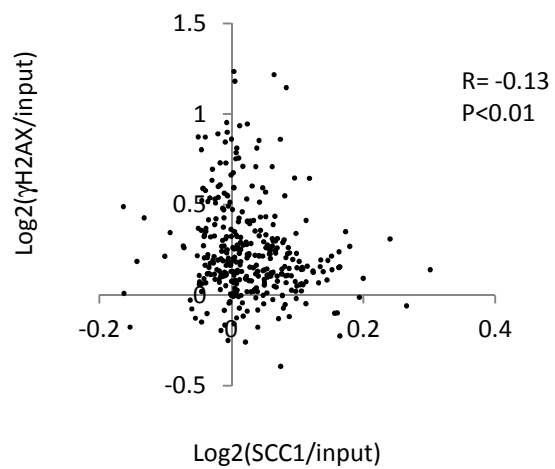

Supplement: Figure S11 — SCC1 counteracts γH2AX. A, Detailed views of the γH2AX/H2AX signal (red) and the SCC1/input signal (black) around an AsiSI site (arrow), expressed as log2 and smoothed using a 500 probes sliding window. B, The average Log2 (γH2AX/input) (y axis) and Log2 (SCC1/input) (x axis) were calculated over the entire length of each of the 359 genes encompassed within γH2AX domains, and plotted against each other. The correlation coefficient and the p value are indicated. (PDF) [file pgen.1002460.s011.pdf]

A

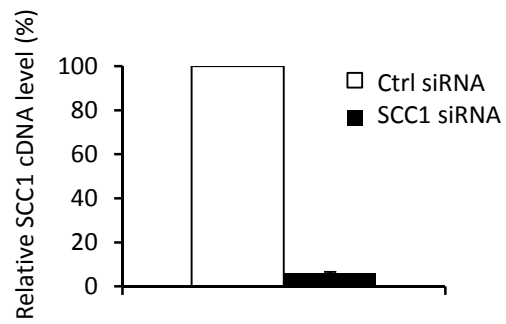

B

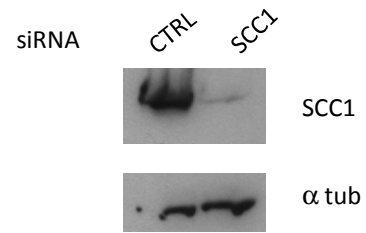

C

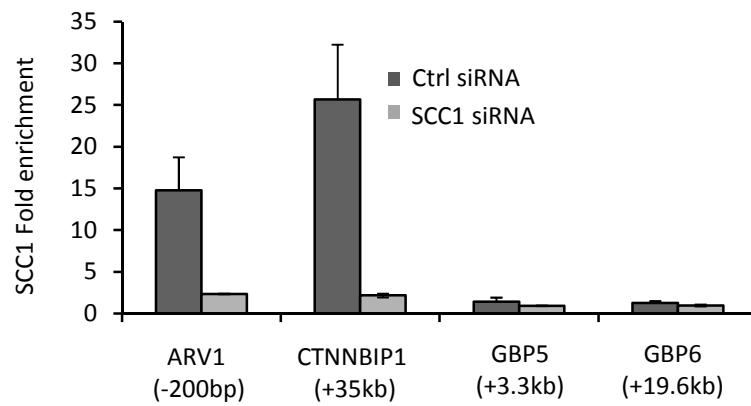

Supplement: Figure S12 — SCC1 is depleted at the RNA level, protein level, and on chromatin upon SCC1 siRNA treatment. A, AsiSI-ER-U20S cells were transfected by electroporation with control (CTRL) or SCC1 siRNA. 48 hours after transfection, mRNA was extracted, reverse transcribed and the amounts of SCC1 and ribosomal phosphoprotein P0 cDNAs were measured by Quantitative real time PCR. SCC1 cDNA levels are shown relative to P0 levels. The mean and standard deviation of the mean (SDOM) from 3 independent experiments are shown. B, SCC1 protein level was analyzed by western blot in Control or SCC1 siRNA transfected AsiSI-ER-U20S cells (using Abcam SCC1 antibody ab992, upper panel). The same blot was also probed for alpha-tubulin as a loading control (lower panel). C, The depletion of chromatin-bound SCC1 by siRNA was analyzed by ChIP in siRNA transfected AsiSI-ER-U20S cells, using an anti SCC1antibody (Abcam ab992) or no antibody. SCC1 enrichment was assessed by Q-PCR on the four regions further described in Figure S22 (two cohesin-bound, and two cohesin-unbound regions). The mean of the relative enrichment of SCC1 over no antibody, from three independent experiments are plotted with SDOM. (PDF) [file pgen.1002460.s012.pdf]

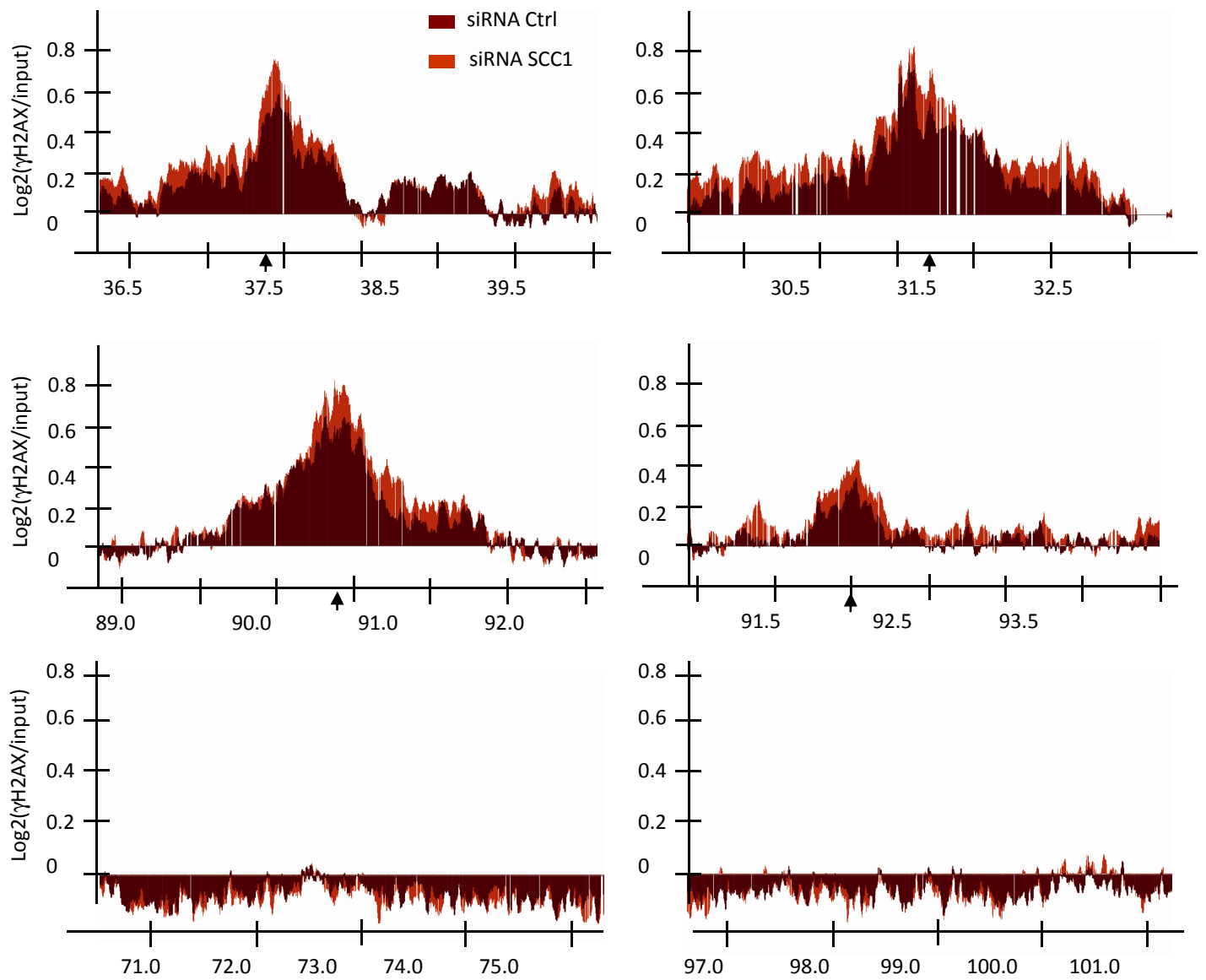

Supplement: Figure S13 — Example γH2AX profiles around AsiSI sites upon SCC1 depletion. Detailed views, around selected AsiSI sites, indicated by arrows (upper and middle panels) and on two genomic regions devoid of AsiSI sites (lower panels). γH2AX enrichment over input in control (dark red) and in siRNA SCC1 (light red) transfected AsiSI-ER-U20S cells, are shown expressed as log2 and smoothed using a 500 probes sliding window. ChIP-chip analysis was performed using chromatin from AsiSI-ER-U20S cells treated with 4OHT. The average of two independent experiments is shown. Note that within domains, the γH2AX signal increases upon SCC1 depletion. Genomic coordinates (x-axis) are indicated in megabase (MB). (PDF) [file pgen.1002460.s013.pdf]

A

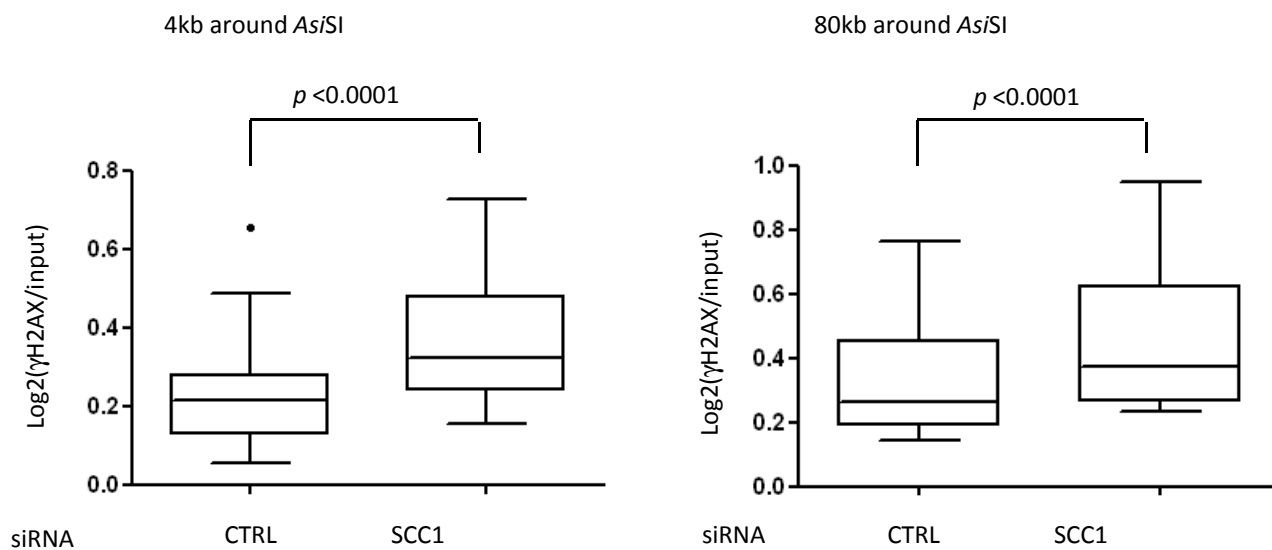

B

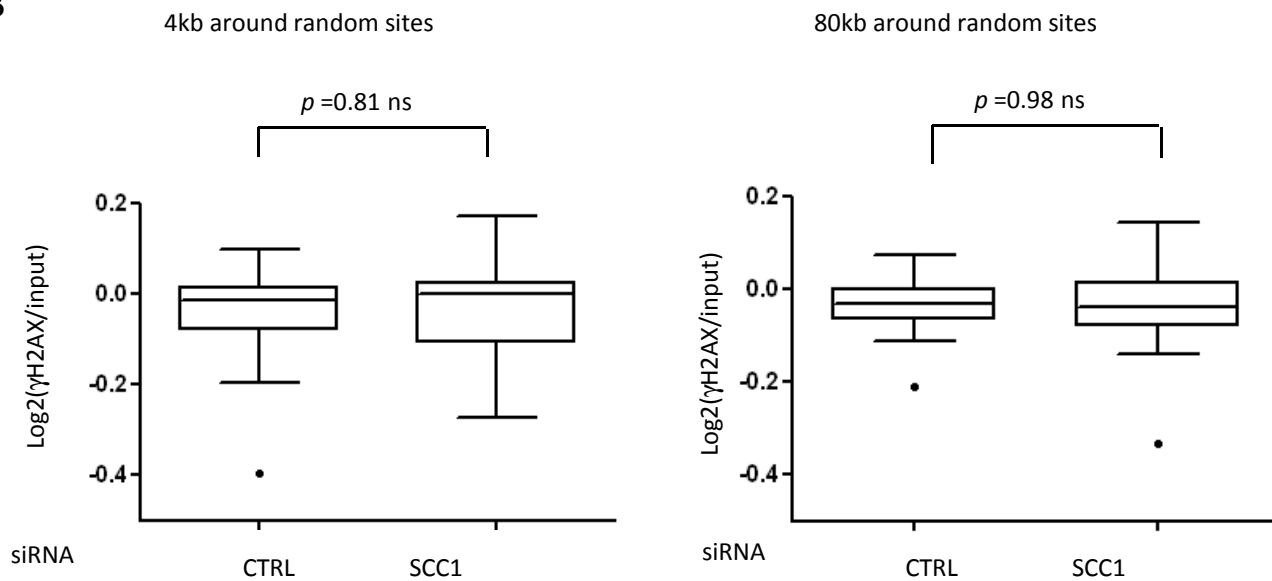

Supplement: Figure S14 — γH2AX increases around DSBs in SCC1 depleted cells. A, The average Log2 (γH2AX/input) was calculated over a 4 kb window (left panel) or an 80 kb window (right panel) surrounding AsiSI sites, in cells transfected with control or SCC1 siRNA as indicated. The box plots represent the distribution of the values obtained for the 24 AsiSI sites. The γH2AX level in SCC1 depleted cells is significantly different from the level observed in control cells. B, Same as in A except that random windows of 4 kb (left panel), and 80 kb (right panel) outside γH2AX domains were averaged. (PDF) [file pgen.1002460.s014.pdf]

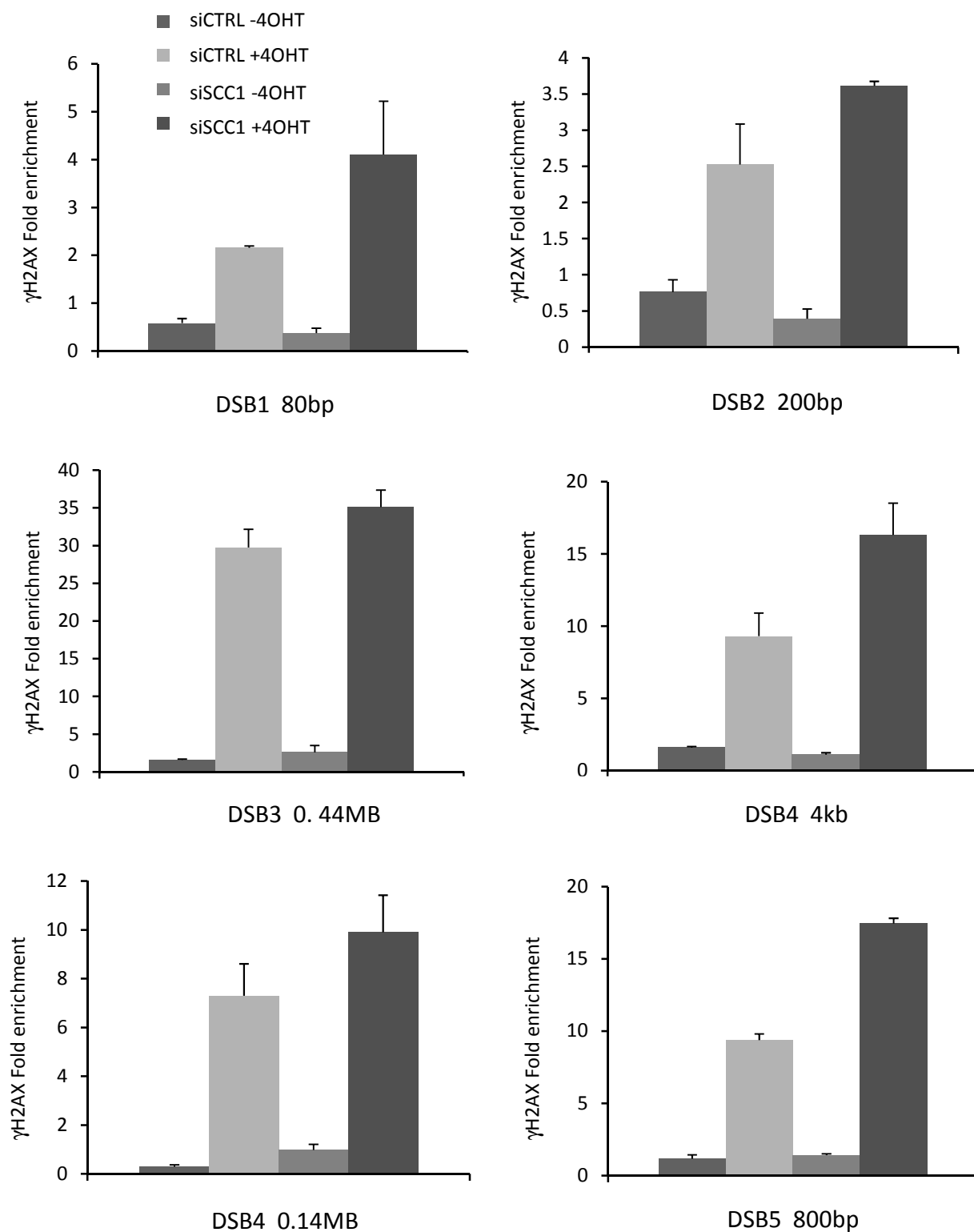

Supplement: Figure S15 — 4OHT-induced γH2AX increases in SCC1 depleted cells compared to control cells. AsiSI-ER-U20S cells were transfected with Control (CTRL) or SCC1 siRNA for 48 hours. Untreated or 4OHT treated cells were subjected to ChIP analyses against γH2AX. Enrichment was scored by Q-PCR within 5 γH2AX domains. Distances of the primers from the DSB are indicated. Data are normalized to the signal observed on a genomic location devoid of DSBs. Representative experiments are shown. (PDF) [file pgen.1002460.s015.pdf]

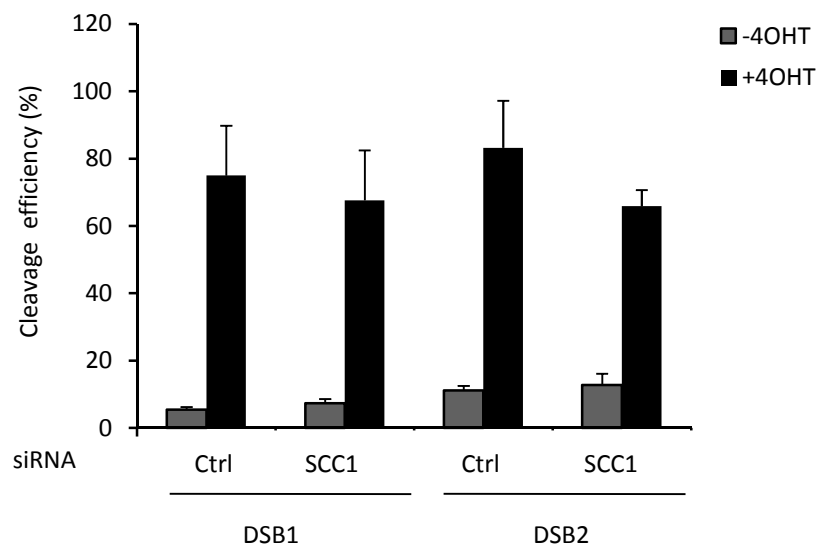

Supplement: Figure S16 — SCC1 depletion does not change the cleavage efficiency of AsiSI sites. Genomic DNA was extracted from siRNA transfected AsiSI-ER-U20S cells treated or not with 4OHT for 4H and assayed for cleavage at AsiSI sites. Pulled down DNA was analyzed by quantitative PCR amplification using primers close to two cleaved AsiSI sites. The mean and standard deviation of the mean (SDOM) from 3 independent experiments are shown. (PDF) [file pgen.1002460.s016.pdf]

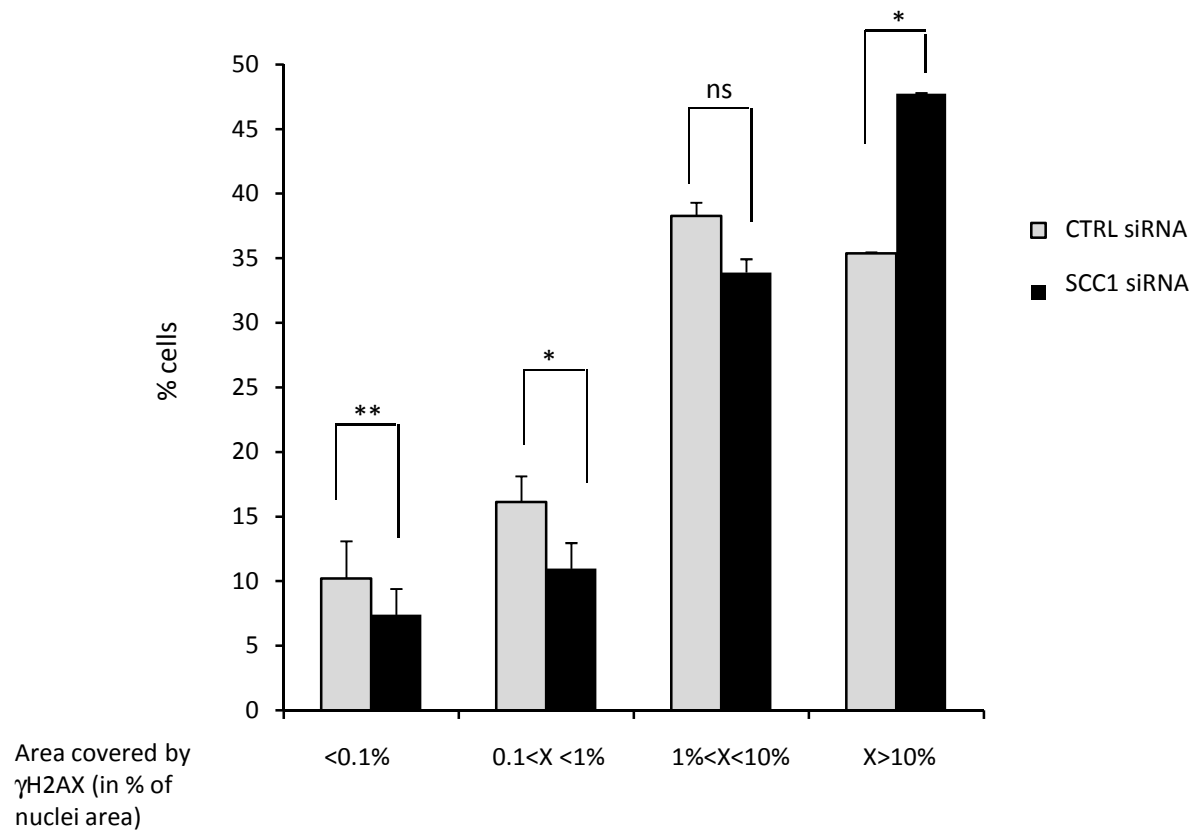

Supplement: Figure S17 — SCC1 depletion leads to an increase of γH2AX as detected by immunofluorescence. AsiSI-ER-U2OS cells transfected with Control (CTRL) or SCC1 siRNA for 48H were treated with 4OHT for 4H and subjected to γH2AX immunofluorescence (Cell Signaling). Images were quantified and classified based on the percentage of their nucleus covered by γH2AX staining. Data are represented as the percentage of cells falling in each of four different categories. The mean and SDOM of three independent experiments are shown. (* p<0.05; ** p<0.01) (PDF) [file pgen.1002460.s017.pdf]

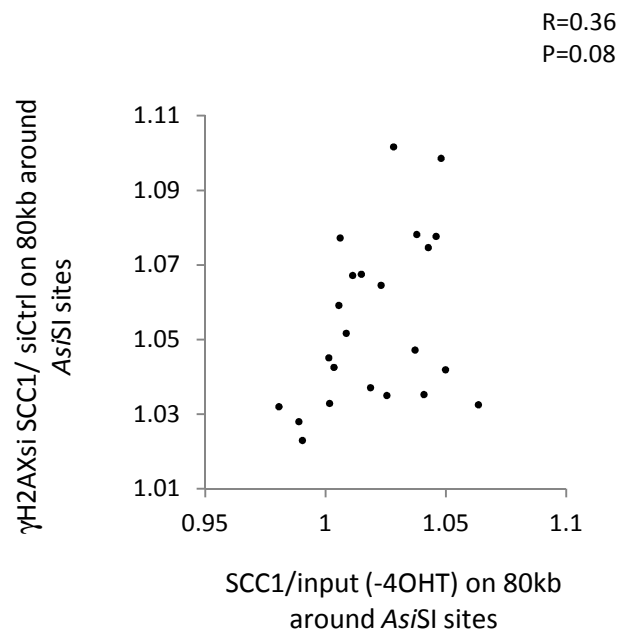

Supplement: Figure S18 — γH2AX increases on SCC1 rich domains upon SCC1 depletion. The averaged SCC1 signal over an 80 kb window around each AsiSI site was calculated (x-axis) and plotted against the γH2AX ratio in cells transfected with siRNA SCC1 versus siRNA CTRL(y axis). Pearson correlation and p value are indicated. (PDF) [file pgen.1002460.s018.pdf]

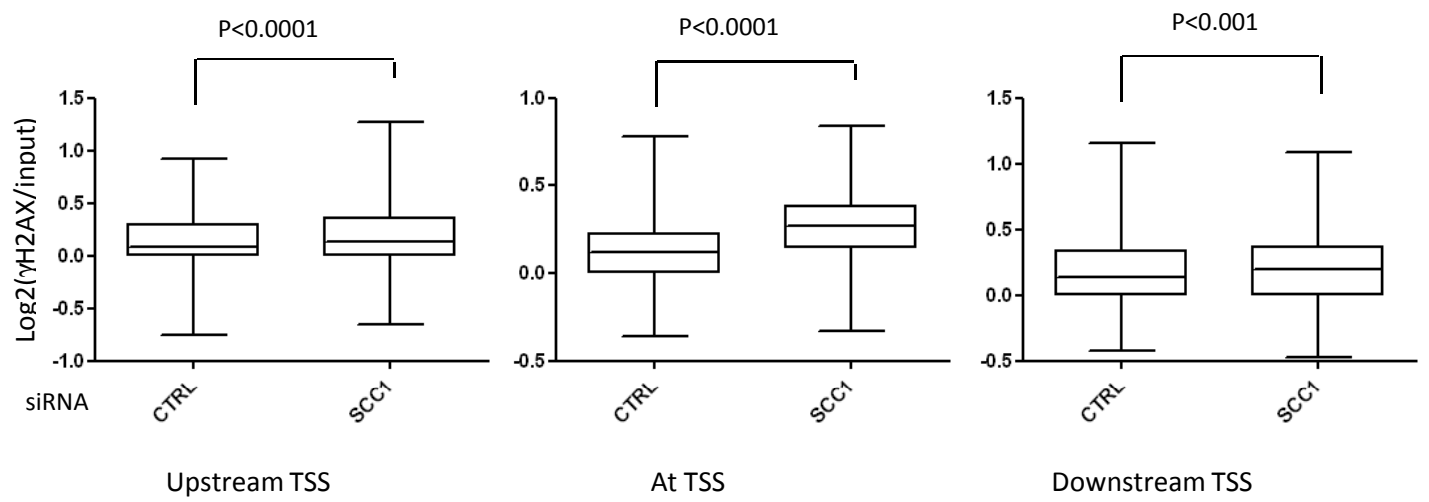

Supplement: Figure S19 — γH2AX increases at TSS upon SCC1 depletion. The average Log2 (γH2AX/input) upstream to promoters (−2000 to −1600 bp) (left panel), at promoters (−200 to +200 bp) (middle panel) and downstream of promoters (+1600 to −2000 bp) (right panel) for the 359 genes encompassed in γH2AX domains were calculated, in Control and SCC1 siRNA transfected cells as indicated. Distributions are represented as box plots. The p values (paired t-test) are indicated above. Note that the biggest increase of γH2AX upon SCC1 depletion occurs at TSSs. While we can see a significant increase both upstream and downstream, it is much weaker than the increase observed at the TSS. (PDF) [file pgen.1002460.s019.pdf]

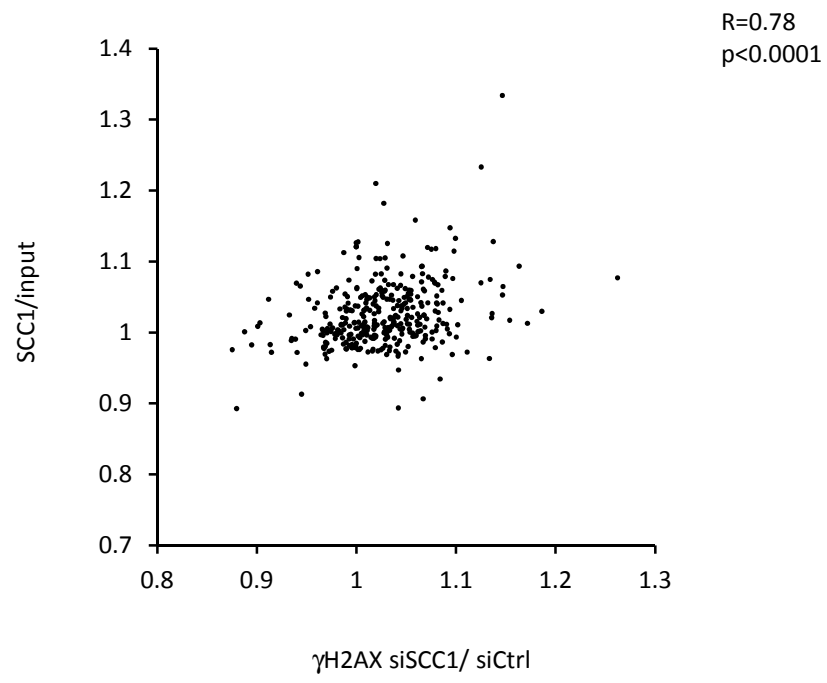

Supplement: Figure S20 — γH2AX increases on SCC1 rich genes upon SCC1 depletion. For each gene encompassed in γH2AX domains, the SCC1 signal was averaged and plotted against the ratio of γH2AX in SCC1 depleted versus control cells. Pearson correlation and p value are indicated. (PDF) [file pgen.1002460.s020.pdf]

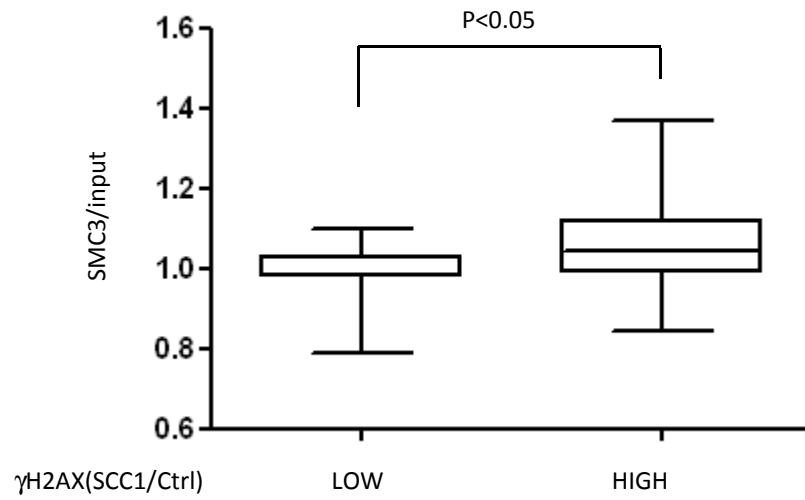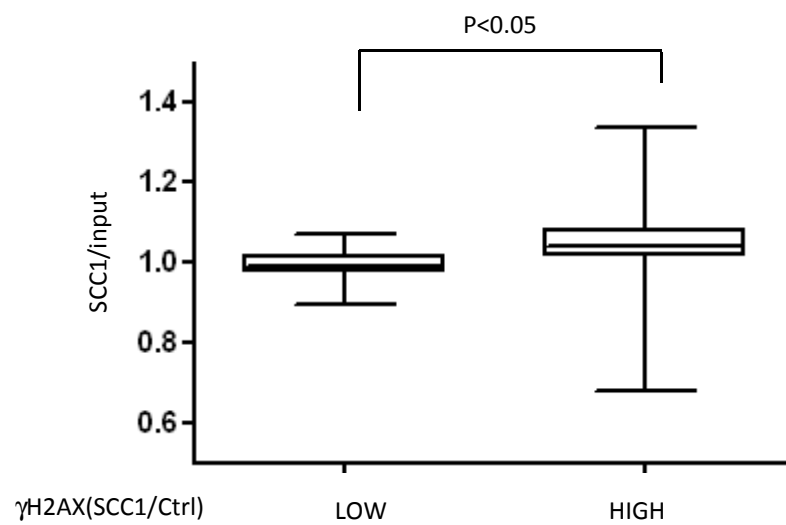

Supplement: Figure S21 — Genes showing high changes in γH2AX between SCC1 siRNA and control cells, show higher levels of cohesin. As for Figure S19, for each gene encompassed in γH2AX domains, the ratio of γH2AX in SCC1 depleted versus control cells and the SMC3 signal (top panel) were averaged. The box plots show the difference in SMC3 (top panel) or SCC1 (bottom panel) between genes showing low (<0.95) and high (>1.1) γH2AX (SCC1/CTRL) ratio. The genes on which γH2AX increases the most after SCC1 depletion, show significantly more SMC3 (upper panel) and SCC1 (lower panel). (PDF) [file pgen.1002460.s021.pdf]

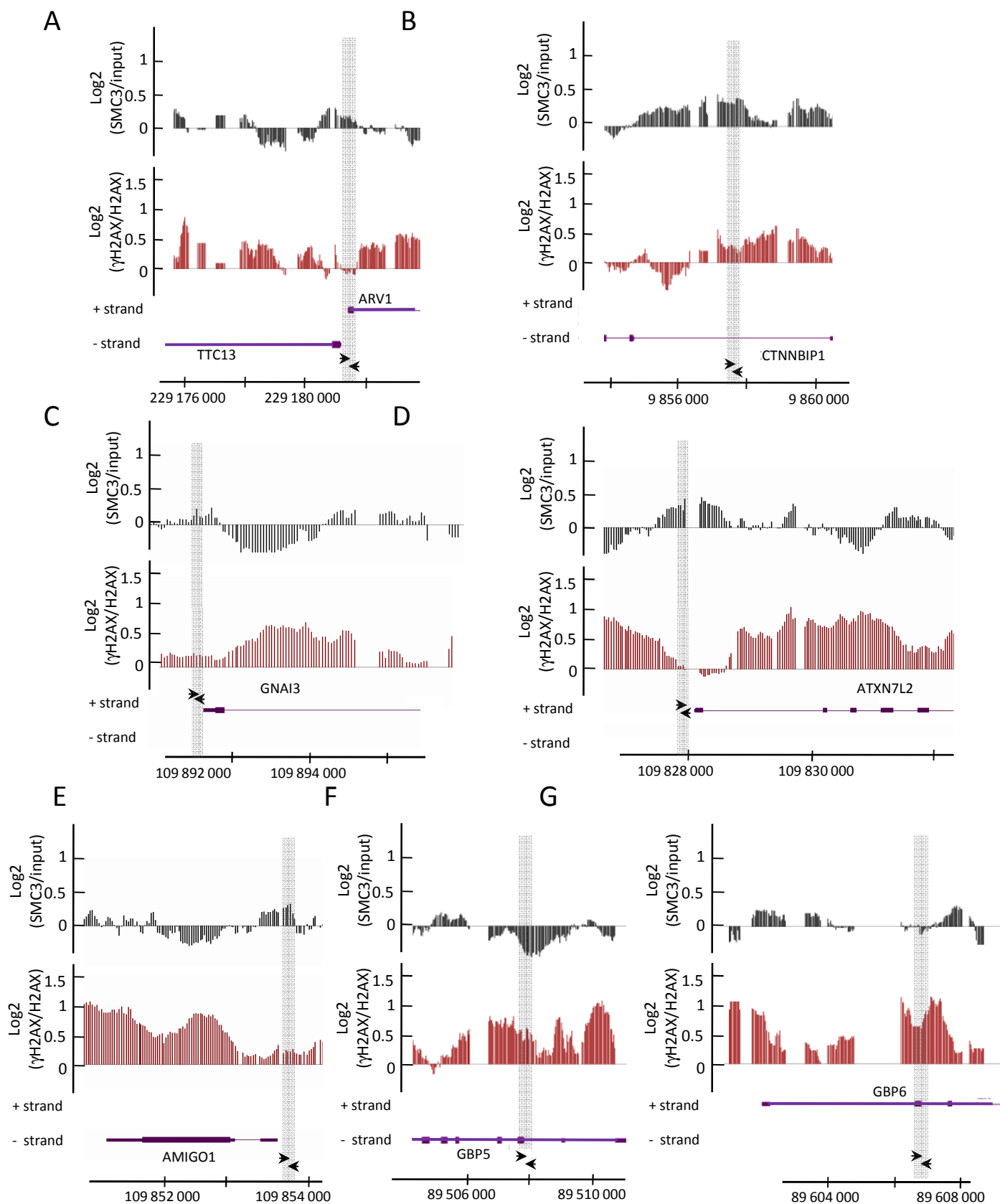

Supplement: Figure S22 — Detailed views of the areas analyzed by Q-PCR with SCC1 siRNA. Detailed views of the SMC3/input (black) and γH2AX/H2AX (red) ChIP-chip data, on five cohesin-bound regions (A–E) and two cohesin-unbound regions (F–G). Positions of the primer pairs used for the Q-PCR analysis presented in Figure 4 are shown (arrows and grey boxes), as well as the position and orientation of genes. (PDF) [file pgen.1002460.s022.pdf]

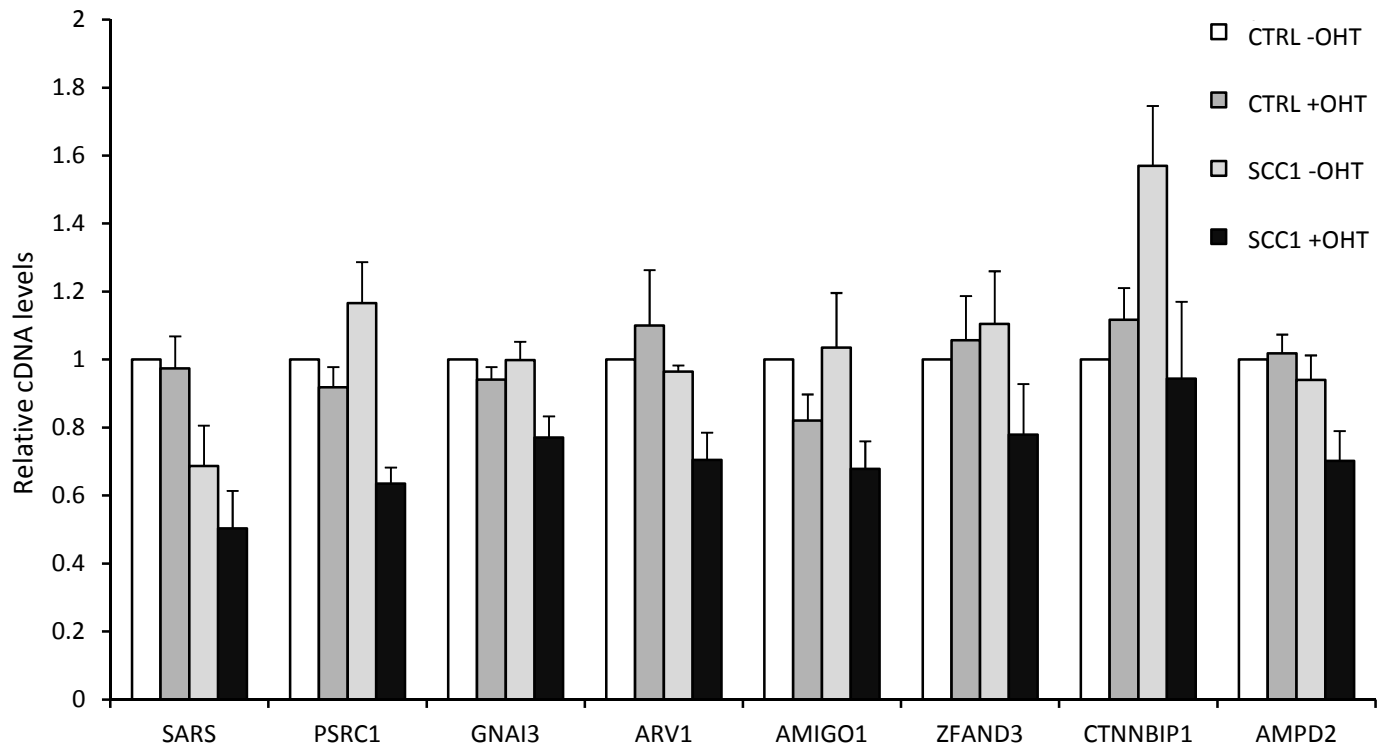

Supplement: Figure S23 — Expression fold changes upon 4OHT treatment and SCC1 depletion. AsiSI-ER-U20S cells were transfected with the indicated siRNAs. After 48 h, cells were treated or not with 4OHT as indicated. Total RNAs were extracted and reverse transcribed. The amount of each cDNA was measured by quantitative real-time PCR, divided by the amount of P0 cDNA and calculated relative to 1 for cells transfected with the control siRNAs and not treated with 4OHT. (PDF) [file pgen.1002460.s023.pdf]

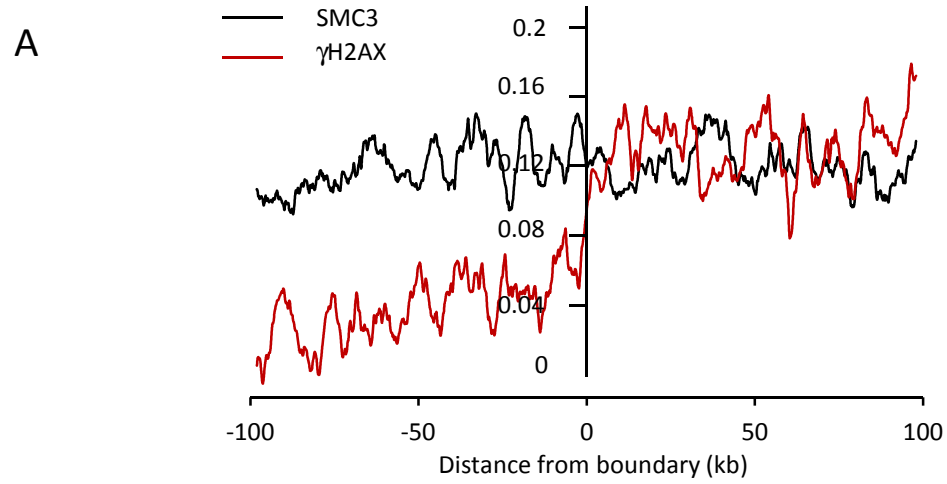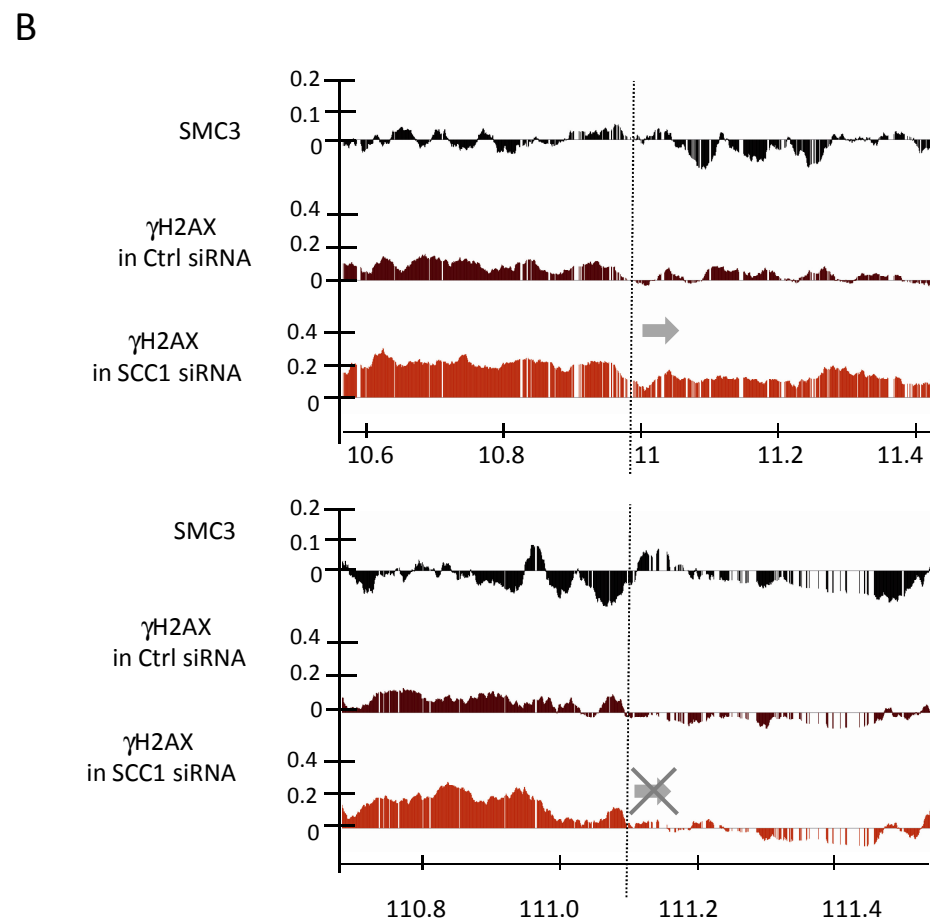

Supplement: Figure S24 — Cohesin binding does not correlate with boundary position. A, Boundaries of γH2AX domains were aligned and overlaid (right and mirror left borders are combined). Data are shown over a 200 kb window centered on domain boundaries and averaged using a 10 kb window size. Profiles are shown for γH2AX (red) and for SMC3 (black) in control cells. Note that we cannot see a specific increase of SMC3 at the boundary. B, Detailed views of right boundaries from two γH2AX domains. Signals for SMC3 (black), γH2AX in control cells (red) and γH2AX in SCC1 depleted cells (orange) are presented. The upper domain shows an extension of γH2AX upon SCC1 depletion, while the lower domain does not (grey arrows). However this is not correlated with the presence of SMC3 at the boundaries (black). (PDF) [file pgen.1002460.s024.pdf]

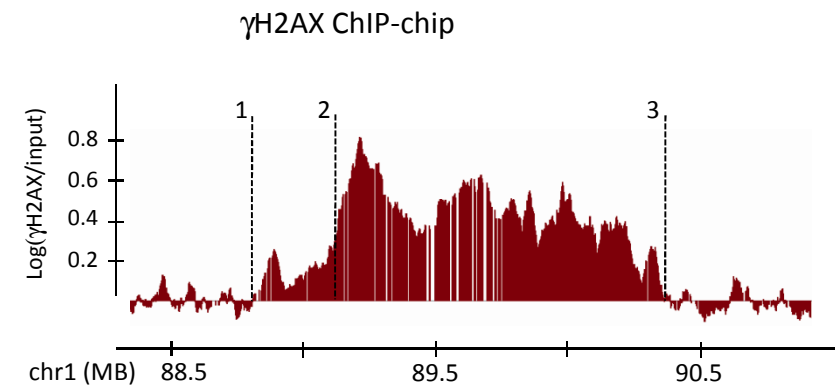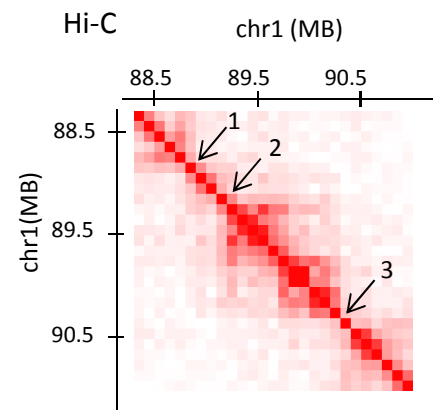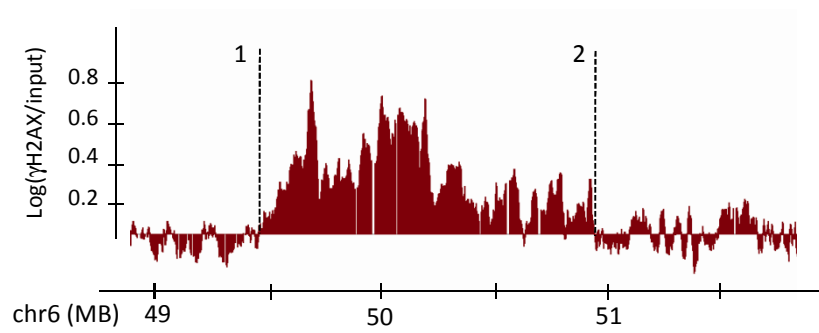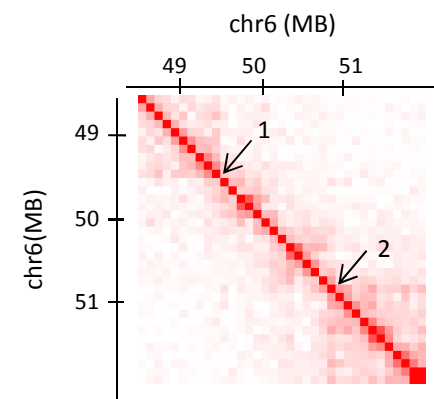

Supplement: Figure S25 — Comparison of γH2AX boundaries with chromosomal domain transitions. Hi-C realized with the human lymphoblastoid cell line GM06990, led to the identification of chromosomal domains at various scales, from the nucleus scale (such as the open and closed chromatin compartments) to a megabase scale. Chromosomal domains could be easily visualized using a heatmap to depict intrachromosomal interactions [52], [57]. The spatial compartmentalization is illustrated by the squared motifs on the heatmap and thus a transition between chromatin domains appears as a “node” between the squares. This level of chromosomal organization only marginally differs between cell lines [52] thus we compared the Hi-C data with our γH2AX profiling data. Inspection of various γH2AX domains, using this representation of loci interaction, showed a clear correlation between our γH2AX domains boundaries in AsiSI-ER-U20S cells and the spatial compartmentalization observed in GM06990 cells (see arrows). (PDF) [file pgen.1002460.s025.pdf]

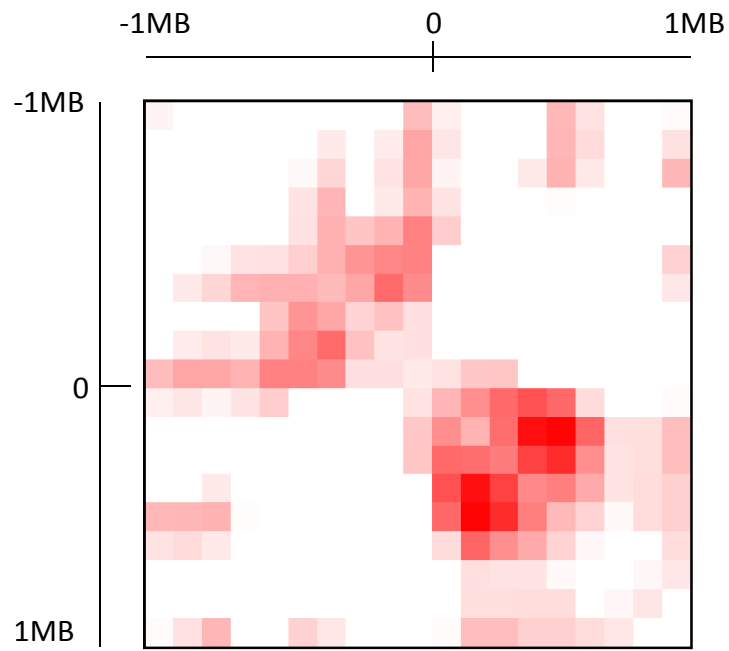

Supplement: Figure S26 — Averaged interaction matrix around γH2AX domain boundaries. The Hi-C interaction matrix [52] located from −1 MB to +1 MB around each identified γH2AX domain boundary (23 AsiSI domains i.e. 46 boundaries) were retrieved and averaged (left and mirror right boundaries were combined). The averaged boundary (the 0 position) correlates with a chromosomal domain transition found using Hi-C. (PDF) [file pgen.1002460.s026.pdf]
