# Supplementary material for: Cohesin Protects Genes against γH2AX Induced by DNA Double-Strand Breaks
Source: PLoS Genet. 2012 Jan 19;8(1):e1002460. doi: 10.1371/journal.pgen.1002460 (PMC3261922; doi:10.1371/journal.pgen.1002460)
Supplement: Table S1 — List of cleaved AsiSI sites on chromosome 1 and chromosome 6. All genomic coordinates are from the genome assembly NCBI Build 36.1. The AsiSI sites efficiently cleaved were determined thanks to our previous analysis using both the γH2AX signal and the cleavage signal [10]. (PDF) [file pgen.1002460.s027.pdf]

Caron et al, Table S1

Chr	beg	end
chr1	9572021	9572029
chr1	9634441	9634449
chr1	14015263	14015271
chr1	19684730	19684738
chr1	25445630	25445638
chr1	40747219	40747227
chr1	89231173	89231181
chr1	91970651	91970659
chr1	109838211	109838219
chr1	202647064	202647072
chr1	206483399	206483407
chr1	222099259	222099267
chr1	229070845	229070853
chr1	240754390	240754398
chr6	20320288	20320296
chr6	27253334	27253342
chr6	27769868	27769876
chr6	31213395	31213403
chr6	37429778	37429786
chr6	50025530	50025538
chr6	90404896	90404904
chr6	135861029	135861037
chr6	144649250	144649258
chr6	149929787	149929795
