# Supplementary material for: Cohesin Protects Genes against γH2AX Induced by DNA Double-Strand Breaks
Source: PLoS Genet. 2012 Jan 19;8(1):e1002460. doi: 10.1371/journal.pgen.1002460 (PMC3261922; doi:10.1371/journal.pgen.1002460)
Supplement: Table S2 — List of γH2AX domain boundaries on chromosome 1 and chromosome 6 determined using the γH2AX signal in siRNA control transfected cells. All genomic coordinates are from the genome assembly NCBI Build 36.1. The boundaries positions were determined using the algorithm described in [10]. (PDF) [file pgen.1002460.s028.pdf]

chr	Beg	End
chr1	8876505	10941655
chr1	13683067	14206694
chr1	14574013	15458248
chr1	18828602	20690157
chr1	25348306	25632473
chr1	40179689	41335924
chr1	88842752	90338214
chr1	91760343	92168228
chr1	108777284	110795366
chr1	202298996	203253833
chr1	206238504	206519991
chr1	228256963	229846958
chr1	240661371	241078266
chr6	20217865	20514974
chr6	27172801	28462497
chr6	29797046	32294998
chr6	36703459	38763996
chr6	49522738	50675025
chr6	89690126	91368262
chr6	135582363	135980869
chr6	144300589	144928926
chr6	149152755	150552914
