# Supplementary material for: Cohesin Protects Genes against γH2AX Induced by DNA Double-Strand Breaks
Source: PLoS Genet. 2012 Jan 19;8(1):e1002460. doi: 10.1371/journal.pgen.1002460 (PMC3261922; doi:10.1371/journal.pgen.1002460)
Supplement: Table S3 — List of primers used in this study. (PDF) [file pgen.1002460.s029.pdf]

Caron et al, Table S3

Primers for Q-PCR

ChIP / Cleavage

ARV1	FW : ACAAGGGGAGAAGCATTGTG	REV : GTTGCCCATTTCCACTCAAC
CTNNBIP1	FW : AGTAGTGGGCCTTGGGCTAT	REV : TGGCCAGGTCCTTGTATTTC
GNAI3	FW : CGGGAGGGCATTATGAGTTA	REV : TCCTCAGGGAGAGGAGTTGA
AMIGO1	FW : AAAGTGAATTCGCTGCACCT	REV : GCCCTTCAGTGGTACCTCAA
ATXN7L2	FW : CCTCCCTGCACCTACCCTAT	REV : TCCACTCTCCCAAGCTCACT
I-SCE-1 (300bp)	FW : GGCCACAAGTTCAGCGTGTC	REV / AAGCACTGCACGCCGTAGGT
I-SCE-1 (2460bp)	FW : CTGGTCATCATCCTGCCTTT	REV : ATGCAGATCGAGAAGCACCT
DSB4_4000bp	FW : CCTTCTTTCCCAGTGGTTCA	REV : GTGGTCTGACCCAGAGTGGT
DSB4_0.14MB	FW : TCAACTCTCCTGCCTCCCTA	REV : CCCTTTGCATATCTCCTCCA
DSB5_800bp	FW : TTCCTGCAGCCTCATTTTCT	REV : TGATGATGCCTTTTCCCTTC
DSB3_0.44MB	FW : GCATCACCTTGTCCCTTCAT	REV : CTATTCGCTCAGCTGCTCCT
GBP5	FW : GCAGACAGGATCTCCAAAGC	REV : CCCCAGGTTCAGTAAGCAAA
GBP6	FW : GGGAGGCTCTTCTAGGTTGG	REV : TGATTCTGTCCTGCCAGATG
No DSB	FW : CCCATCTCAACCTCCACACT	REV : CTTGTCCAGATTCGCTGTGA
DSB1_80bp	FW : GATTGGCTATGGGTGTGGAC	REV : CATCCTTGCAAACCAGTCCT
DSB2_200bp	FW : TGCCGGTCTCCTAGAAGTTG	REV : GCGCTTGATTTCCCTGAGT
Cohesin Binding Site	FW : CAGCTCTGTGTCCTGTCTTATCC	REV : CAGCTATAATTGATGAAGAGGCG
GAPDH	FW : GAAGGTGAAGGTCGGAGTCA	REV : GAAGATGGTGATGGGATTTC
DSB1 _319bp	FW: AGGACTGGTTTGCAAGGATG	REV: ACCCCCATCTCAAATGACAA
DSB1_500bp	FW: CCTGGATATGAGTTTGATCAGC	REV: CTCTCCTTTCGCTGACACTG
DSB1_1019bp	FW: AGGAATTGACTGCGGTGTTC	REV: GGGGAGGAGGAAAGGTGTAG
DSB1_2500bp	FW: GCCATAACAGAGGGTGGAAA	REV: AACTTTAGGATGGGGCTGCT
DSB1_3200bp	FW: ACATGGGTCTTCCAGGTGAC	REV: GGAACTTACAACCCCACACTT
DSB3_41kb	FW: GCATCACCTTGTCCCTTCAT	REV: CTATTCGCTCAGCTGCTCCT
DSB3_0.78Mb	FW: GCTCTTCCCTGTGAGGTCTG	REV: CGATGAAACAGGCAAATGA
DSB3_1.14Mb	FW: GCATGCATCTAGGGGTCACT	REV: AGGTTGCATAGGGCAACTCA
DSB3_1.42Mb	FW: GGAAGTCAGCCGTGAGACC	REV: AGCGTCCCTCGAAGTACAAA
DSB3_1.45Mb	FW: GCTAGAGGCTCTGCTCTGGA	REV: GGGGTATCTAGCCCTCGTTC
DSB3_1.49Mb	FW: ATGTGTTCTTTGCGACACCA	REV: TGCAAGGGAAGAAAATGTCC

Caron et al, Table S3

Primers for Q-PCR

RT

SARS	FW : CTGGCCTGTCTACCTGCTTC	REV : CTGGCAGCATGATTCAAAGA
PSRC1	FW : GGGAAAAGAGGTCACCTTCC	REV : AAGCAGGCCAGAGTCCAGTA
GNAI3	FW : GGGGTACAAGCTTGCTTCAG	REV : TTGGCCACCTACATCAAACA
ARV1	FW : AACCAGGAGGCCAAAGAGTT	REV : CCACCACCTCAGGTATGCTT
AMIGO1	FW: CCAAGTAATGAACGGGTGCT	REV: CTGTGTTGAGGGTGTCATGG
ZFAND3	FW: GGAGGAAGCCATCATGAAAA	REV: TGGCTGGCTAAAGAAAGGAA
AMPD2	FW: CGTAGTGCCCCGTATGAGTT	REV: CGAGTCACTGTCCGTCTTCA
CTNNBIP1	FW : TTGGCTGCAGGAAGAAACTT	REV : GCAGCCAATCAGACCTCTTC
P0	FW : GGCGACCTGGAAGTCCAACT	REV : CCATCAGCACCACAGCCTTC
SCC1	FW : TGACTTTGATCAGCCACTGC	REV : TCTCACGATCATCCATTCCA

siRNA

siSCC1	FW : GGUGAAAAUGGCAUUACGGtt	REV : CCGUAAUGCCAUUUCACCtt
siCtrl	FW : CAUGUCAUGUGUCACAUCUtt	REV : AGAUGUGACACAUGACAUGtt

Biot-oligo

Test Cleavage	FW : CGCAAGCTTTAATACGACTCACTATAGGG	REV : Biot-CCCTATAGTGAGTCGTATTAAAGCTTGCGAT
